# Spatial Domain‐Based Approach to Analyze the Mechanism of Sparganii Rhizoma‐Curcumae Rhizoma Pair in the Treatment of Colorectal Cancer

**DOI:** 10.1002/fsn3.70794

**Published:** 2025-08-19

**Authors:** Yuan‐jie Liu, Qian‐wen Ye, Yi‐lan Jin, Qian Zhang, Xi Zou, Jie‐pin Li

**Affiliations:** ^1^ Department of Oncology Affiliated Hospital of Nanjing University of Chinese Medicine, Jiangsu Province Hospital of Chinese Medicine Nanjing Jiangsu China; ^2^ Department of Oncology Affiliated Hospital of Jiangxi University of Chinese Medicine, Jiangxi Province Hospital of Chinese Medicine Nanchang Jiangxi China; ^3^ Department of Dermatology Zhangjiagang TCM Hospital Affiliated to Nanjing University of Chinese Medicine Zhangjiagang Jiangsu China; ^4^ Key Laboratory of Tumor System Biology of Traditional Chinese Medicine Nanjing Jiangsu China

**Keywords:** Curcumae Rhizoma, network pharmacology, Sparganii Rhizoma, spatial domain

## Abstract

Colorectal cancer (CRC) is a global health challenge shaped by a complex tumor microenvironment (TME), where myeloid‐driven inflammatory and immunosuppressive processes play central roles. Understanding the spatial and dynamic interactions within the TME is essential for uncovering novel regulatory mechanisms. We integrated spatial transcriptomics, single‐cell RNA‐sequencing, and network pharmacology to develop a comprehensive analytical framework for deconstructing cellular and molecular interactions across spatial domains within the TME. Using this approach, we investigated the potential mechanisms by which the Sparganii Rhizoma‐Curcumae Rhizoma (SCP) Chinese medicine (TCM) herbal pair may influence primary CRC and liver metastases. Our analysis suggests that in primary CRC, SCP may affect neutrophil‐enriched domains through modulation of CEACAM‐related signaling, while in liver metastases, it may influence macrophage‐dominated domains associated with APP signaling. These findings highlight possible domain‐specific molecular interactions and suggest that SCP may regulate intra‐ and inter‐domain signaling patterns relevant to TME remodeling. This study establishes a novel integrative methodology to investigate spatial domain‐specific mechanisms within the TME. Our findings reveal the multi‐dimensional effects of SCP, providing a precision‐targeted, TME‐centric therapeutic strategy for CRC. The framework aligns with TCM principles and offers new avenues for personalized cancer therapies.

## Graphical abstract

A novel framework integrates single‐cell, spatial, and network pharmacology methods. SCP targets CEACAM19 to modulate neutrophils in primary tumors and targets APP to modulate macrophages in liver metastases. SCP restructures intra‐ and inter‐domain signaling networks to regulate tumor progression.

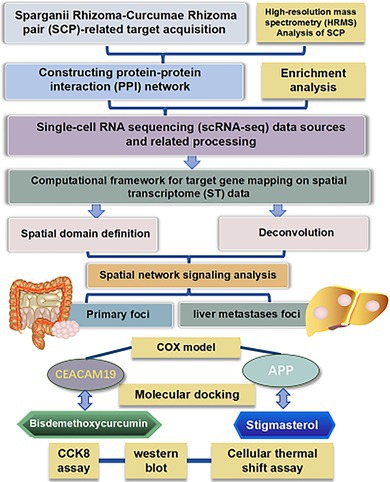

## 1 Introduction

CRC remains the third most prevalent cancer globally, with an estimated 1.92 million new cases and 930,000 deaths in 2020 (Siegel et al. 2023; Morgan et al. 2022). Current treatment strategies include surgery, chemotherapy, targeted therapies (e.g., bevacizumab), and immunotherapy (Benson et al. 2018; Hansen et al. 2021). However, adverse effects like hypertension, gastrointestinal perforations, and the high cost of these therapies limit their accessibility and tolerability (Lopez et al. 2019; Abdel‐Qadir et al. 2017). Ongoing research is essential to develop more personalized treatments with fewer side effects.

Recent studies have increasingly recognized the role of Traditional Chinese Medicine (TCM) in the treatment of CRC, particularly in reducing chemotherapy side effects and improving patient outcomes (Sun et al. 2021). TCM is valued for its low cost and long history of use in cancer care, with herbal formulas showing potential in alleviating symptoms and enhancing chemotherapy efficacy. Its affordability and effectiveness make it a valuable adjunct to conventional therapies (Zhao et al. 2024; Yang, Zhou, et al. 2021). In TCM, blood stasis and qi stagnation are considered key factors in the development of CRC (Yang et al. 2019; Jin et al. 2022), as these conditions disrupt normal physiological functions and promote the formation of an inflammatory microenvironment conducive to tumor growth. Qi stagnation leads to blood stasis, which causes poor circulation and oxygen supply, further exacerbating local inflammation and enhancing tumor aggressiveness (Qing‐Hua et al. 2010). The inflammatory response triggered by blood stasis and qi stagnation promotes the release of various pro‐inflammatory cytokines, fostering tumor cell proliferation, invasion, and metastasis (Xiao 2012). TCM treatments focus on improving these conditions by invigorating blood circulation and promoting qi flow. This approach aims to restore normal intestinal function, reduce cancer toxicity, improve the tumor microenvironment, and alleviate chemotherapy side effects.

Sparganii Rhizoma (SR) and Curcumae Rhizoma (CR), as a medicinal pair, hold significant therapeutic importance in the treatment of CRC (Lin et al. 2024), particularly in relation to the TCM theory of “qi stagnation and blood stasis.” SR improves the tumor microenvironment and promotes blood circulation through its blood‐activating and stasis‐removing properties, while CR inhibits tumor growth and metastasis through its ability to regulate qi, break blood stasis, detoxify, relieve pain, and provide anti‐angiogenic and anti‐inflammatory effects (Xu et al. 2015; Zhang et al. 2021). Together, they work synergistically, enhancing treatment efficacy through multiple targets and pathways, reducing tumor proliferation, migration, invasion, and chemotherapy side effects, and improving drug bioavailability and stability (Wu et al. 2024; Liu et al. 2021; Wei et al. 2022). Furthermore, they help regulate qi and blood, unblock meridians, boost immunity, and improve the tumor microenvironment, thereby promoting patient recovery.

Network pharmacology is a key tool for studying traditional TCM formulas, but it faces challenges such as inconsistent data quality, static networks, and limited causal analysis, hindering clinical application (Zhao et al. 2023; Zhang, Zhang, Zhou, et al. 2023). Schürch et al. (Schürch et al. 2020) proposed Cellular Neighborhood (CN), which analyzes spatial‐functional cellular communities in the tumor microenvironment (TME), revealing links between tumor heterogeneity, survival, and treatment response. This approach expands from local cellular functions to overall tissue behavior, overcoming the limitations of traditional methods based on single variables like gene expression or cell proportions. By identifying immune cell aggregation patterns and functional differences between the tumor core and boundary, the CN approach offers insights into tumor progression dynamics and intercellular communication within the TME. In this study, we introduce a novel integration of CN analysis with network pharmacology, aiming to explore how Sparganii Rhizoma and Curcumae Rhizoma (SCP) may influence specific cellular communities relevant to colorectal cancer. This framework extends beyond the traditional binary “compound–target” paradigm, enabling a more contextualized understanding of TCM interventions within spatially organized tumor environments. By incorporating spatial heterogeneity and dynamic cell–cell interactions, our approach provides a foundation for higher‐dimensional investigation into the mechanistic basis of TCM in oncology.

## 2 Materials and Method

### 2.1 Sparganii Rhizoma‐Curcumae Rhizoma Pair (SCP)‐Related Target Acquisition

The active ingredients and a subset of the protein targets of SCP were retrieved from the Traditional Chinese Medicine Systems Pharmacology Database (TCMSP) (https://old.tcmsp‐e.com/tcmsp.php), using predefined criteria of Drug‐likeness (DL) ≥ 0.17 and Oral Bioavailability (OB) ≥ 30%.

Target prediction for Sparganii Rhizoma‐Curcumae Rhizoma pair (SCP) active compounds was performed using three complementary computational approaches. Canonical SMILES strings of SCP compounds meeting drug‐likeness criteria (OB ≥ 30%, DL ≥ 0.17) obtained from the PubChem database were submitted to SwissTargetPrediction (http://www.swisstargetprediction.ch/) (organism: *Homo sapiens* ; probability threshold > 0.1), which employs a combined 2D/3D similarity method against curated ligand‐protein databases. Parallel submissions were made to the Similarity Ensemble Approach (https://sea.docking.org/) (SEA; E‐value threshold < 1e‐5, Tanimoto coefficient > 0.7), which statistically evaluates target associations based on chemical similarity to bioactive ligands in ChEMBL (https://www.ebi.ac.uk/chembl/). Additionally, TargetNet (http://targetnet.scbdd.com/) predictions were executed using its default random forest model (probability threshold > 0.5), which leverages molecular fingerprint descriptors for proteome‐wide affinity estimation. The union of predicted targets from all three platforms underwent identifier standardization via UniProt, followed by removal of duplicate entries to generate a consolidated target list for subsequent validation and analysis.

### 2.2 Constructing Protein–Protein Interaction (PPI) Network

The therapeutic targets associated with SCP were analyzed using the STRING database (Search Tool for the Retrieval of Interacting Genes/Proteins) (https://cn.string‐db.org/) to construct the Protein–Protein Interaction (PPI) network, with the confidence greater than 0.4 to ensure reliable interaction data. The interactions retrieved from STRING represent both direct (physical) and indirect (functional) associations derived from experimental data, computational predictions, and publicly available databases. Subsequently, the resultant PPI network was imported into Cytoscape software (version 3.10.2) (Shannon et al. 2003) for visualization and further topological analysis, enabling a more intuitive exploration of the interaction relationships and the identification of potential hub proteins for further study.

### 2.3 Enrichment Analysis

The therapeutic targets associated with SCP were subjected to functional enrichment analysis using the “clusterProfiler” package in R (Wu et al. 2021). This package was utilized to perform Gene Ontology (GO) and Kyoto Encyclopedia of Genes and Genomes (KEGG) pathway annotations. The GO analysis provided insights into the biological processes, molecular functions, and cellular components related to the SCP targets, while the KEGG pathway analysis highlighted the specific biochemical pathways and cellular signaling pathways potentially modulated by SCP. These analyses were conducted to identify key functional categories and pathways that may play significant roles in the therapeutic effects of SCP.

### 2.4 Bulk Data Sources and Related Processing

Transcriptome information from bulk RNA‐sequencing (RNA‐seq) and corresponding clinical data for 641 patients diagnosed with colon adenocarcinoma (COAD) and rectum adenocarcinoma (READ) were obtained from the TCGA portal. Using R software, the gene expression data were exported in a standardized format as a data matrix.

For further analysis, details are provided below. Pearson correlation coefficients were used to assess variable correlations. The Mann–Whitney U‐test was applied to non‐normally distributed variables. One‐way ANOVA was used for comparing two or more groups. Survival analysis was conducted using the log‐rank (Mantel‐Cox) test. Univariate Cox regression was employed to identify independent prognostic factors, with hazard ratios (HRs) reported. All statistical analyses were performed in R, with statistical significance set at *p* < 0.05 (two‐tailed).

### 2.5 Single‐Cell RNA‐Sequencing (scRNA‐Seq) Data Sources and Related Processing

The scRNA‐seq data involved in this study, including GSE183916 (Lenos et al. 2022), GSE163974 (Wang et al. 2021), GSE217774 (Barrett et al. 2013), GSE231559 (Hsu et al. 2023), and E‐MTAB‐8410 (Lee et al. 2020), were downloaded from the Gene Expression Omnibus portal. We merged GSE183916, GSE163974, GSE217774, and GSE231559 and removed the batch effect using the “IntegrateLayers” function in Seurat V5 (CCA method) (Satija et al. 2015); and obtained the results including peritoneal metastases, liver metastases, primary tumors, normal intestinal tracts, normal intestine, and normal liver tissues in a merged dataset. In addition, the E‐MTAB‐8410 dataset containing tumor location information was processed separately.

For basal processing, the Seurat standard process was followed to calculate UMAP coordinates for individual cells. Each cell was then manually annotated using recognized genetic markers, and the “ggplot2” package (Ito and Murphy 2013) was used to visualize the clustering and annotation results. In order to compare the expression differences of SCP targets in different cell groups, we used the “AddModuleScore” (Liu et al. 2023) function in Seurat to calculate the target scores and the “VlnPlot” and “DotPlot” functions for visualization. In addition, feature plots were generated using the “Nebulosa” package for easy reading.

### 2.6 Computational Framework for Target Gene Mapping on Spatial Transcriptome (ST) Data

The ST data involved in this study, including four colorectal tumors (CT), (CT1: VISDS000775, CT2: VISDS000776, CT3: VISDS000777, and CT4: VISDS000778) and 2 liver metastatic tumors (LM), (LM1: VISDS001055 and LM2: VISDS001056) samples, were downloaded from the CROST database (Wang, Wu, et al. 2024).

Spatial transcriptomics (ST) data processing commenced with preprocessing using the “Seurat” package (v5.0). Raw gene expression matrices were normalized via “SCTransform” function to generate variance‐stabilized counts, followed by dimensionality reduction “RunPCA” function.

Subsequently, the “SPACET” package (v0.1.0) was employed for reference‐free deconvolution using “SpaCET.deconvolution” function with default parameters. This method leverages pan‐cancer gene expression signatures (without paired scRNA‐seq) to estimate cell‐type proportions per spot via non‐negative matrix factorization (NMF), generating a cell proportion matrix.

Spatial domain segmentation was performed using BayesSpace (v1.6.0). We applied “spatialPreprocess” for log‐normalization and PCA, followed by “qTune” to determine optimal clusters. Spatial domains were identified via “spatialCluster” (*q* = 8 for CT/LM samples, *γ* = 3, nrep = 10,000) using a Markov Random Field model that incorporates spatial coordinates.

These outputs were integrated via SpaTopic (v0.1.0). The “CellTopic” function merged BayesSpace‐defined domains with SPACET‐derived cell proportions. Cell‐type enrichment within domains was then quantified, ranking cell types by mean proportion to identify dominant constituents.

Finally, SCP target gene signatures were mapped to ST data using Seurat's “AddModuleScore” function. Enrichment scores were calculated per spot using default parameters (ctrl = 100 genes, binning by expression). Domain‐specific targeting was determined by comparing mean signature scores across spatial domains (threshold: > 90th percentile vs. background). Notably, MOL000915 was excluded from a subset of studies because it was not indexed in the PubChem database and the number of predicted targets was extremely limited, which prevented reliable scoring or meaningful interpretation in target prediction and network analysis. Besides, we studied the non‐random expression patterns of genes associated with the domains targeted by SCP.

To analyze domain‐domain communications, we utilized the ligand‐receptor information from the “CellChat” package and performed the associated analysis and visualization.

### 2.7 Immunohistochemistry (IHC) Image Acquisition

High‐resolution IHC images for CEACAM19 and APP were programmatically retrieved from the Human Protein Atlas (HPA) using the HPAanalyze R package (v1.0). The “hpaDownload” function was executed with parameters hpa_id = c (“CEACAM19,” “APP”), dataType = “ihc,” and resolution = “high” to target protein‐specific IHC data. Image metadata were first queried via “hpaQuery” function using Ensembl gene IDs (ENSG00000105371 for CEACAM19; ENSG00000142192 for APP), followed by filtered retrieval of normal colon and colorectal cancer tissue specimens. The “hpaDownload” function subsequently fetched 4000 × 3000‐pixel TIFF images at 20× magnification using HPA's REST API (https://www.proteinatlas.org/api). All images were downloaded in batch mode with rate limiting (delay = 1 s between requests) to comply with HPA server policies (Tran et al. 2019).

### 2.8 Molecular Docking

Molecular docking simulations were conducted using CB‐Dock2 (https://cadd.labshare.cn/cb‐dock2/php), which is based on AutoDock Vina with a blind docking approach. Default settings were applied for all parameters. The structure of the SCP active ingredient was retrieved from PubChem, while the 3D protein structures of Carcinoembryonic antigen‐related cell adhesion molecule 19 (CEACAM19) and Amyloid beta precursor protein (APP) were predicted using AlphaFold3 (Roy and Al‐Hashimi 2024). This approach enabled the investigation of potential drug–protein interactions by analyzing the binding affinity and conformational compatibility between the active ingredient and the target proteins.

### 2.9 Preparation and Q‐Orbitrap High‐Resolution Mass Spectrometry (HRMS) Analysis of SCP

To verify the chemical presence of active compounds identified via network pharmacology, we performed high‐resolution mass spectrometry (HRMS) analysis of SCP. Take 450 g each of Sparganii Rhizoma and Curcumae Rhizoma, free from mold or impurities. Wash quickly, then slice into 2–3 mm thin slices or 1–2 cm segments. Soak in 8–10 times the volume of water for 40–60 min, then transfer to a clay pot. Boil over high heat, simmer on low for 30–40 min, stirring occasionally, and collect the first decoction. Add 1/2 to 2/3 of the first decoction's volume of water to the residue, simmer for 20–30 min, and filter both decoctions using a 100–200 mesh filter. Combine and concentrate until the desired consistency over low heat. Store in sterile containers, refrigerated at 2°C–8°C for short‐term use or −20°C for long‐term. Shake well after thawing. For analysis, SCP decoction (100 μL) was mixed with 300 μL methanol, vortexed for 10 min, and centrifuged at 20,000 × *g* for 10 min at 4°C. The supernatant was analyzed using a Q Exactive Orbitrap LC–MS/MS system (Thermo Fisher Scientific) with an AQ‐C18 column (150 × 2.1 mm, 1.8 μm). The mobile phase was 0.1% formic acid in water (A) and methanol (B) at a flow rate of 0.3 mL/min. Gradient elution was performed (98% A to 5% A over 20 min, 30 min total runtime), with a 5 μL injection volume at 35°C. Mass data were acquired in positive/negative ion switching modes (100–1500 m/z) at a resolution of 70,000 (Full MS) and 17,500 (MS2), with a spray voltage of 3.2 kV, capillary temperature at 300°C, sheath gas 40, and auxiliary gas 15 at 350°C.

### 2.10 Cell Lines and Culture Conditions

SW480 cells (human primary colorectal cancer cell line, purchased from Pu‐nuo‐sai Life Technology Co. Ltd., Wuhan, China, Cat: CL‐0223) were cultured in Dulbecco's Modified Eagle Medium (DMEM) supplemented with 10% fetal bovine serum (FBS) and 1% penicillin/streptomycin (P/S) at 37°C with 5% CO_2_.

HL‐60 cells (human acute promyelocytic leukemia cell line, purchased from Wuhan Pu‐nuo‐sai Life Technology Co. Ltd., Wuhan, China, Cat: CL‐0110) were cultured in Iscove's Modified Dulbecco's Medium (IMDM) supplemented with 20% FBS and 1% P/S at 37°C with 5% CO_2_. Granulocytic differentiation was induced by treating HL‐60 cells with 1.25% DMSO (Sigma Aldrich, St. Louis, MO, United States, Cat: D2650‐100ML) for 5 days.

KM12‐SM cells (human colorectal cancer liver metastatic cell line, purchased from Zhejiang Meisen Cell Technology Co. Ltd., Zhejiang, China, Cat: CTCC‐001‐0315) were cultured in RPMI‐1640 medium supplemented with 10% FBS and 1% P/S at 37°C with 5% CO_2_.

THP‐1 cells (human monocytic leukemia cells, purchased from the China Academy of Sciences, Shanghai, China, Cat: TCHu 57) were cultured in RPMI‐1640 medium supplemented with 10% FBS and 1% P/S at 37°C with 5% CO_2_. Differentiation into macrophages was induced by treating THP‐1 cells with 100 ng/mL phorbol 12‐myristate 13‐acetate (PMA) (MedChemExpress, USA, Cat: HY‐18739‐1) for 24 h; followed by a 24‐h resting period in fresh RPMI‐1640 medium.

### 2.11 Non‐Contact Co‐Culture Model

To simulate the in situ microenvironment of primary colorectal cancer (CRC) and CRC liver metastasis, a non‐contact co‐culture system was established using Transwell inserts (0.4 μm pore size) in standard 6‐well plates. This setup allows for paracrine signaling through soluble factors without direct cell‐to‐cell contact.

For the primary CRC model, SW480 cells were seeded in the upper chamber at a density of 2 × 10^5^ cells per well. Differentiated HL‐60 cells (granulocytes, 1.25% DMSO‐induced for 5 days) were seeded in the lower Transwell insert at a density of 1 × 10^6^ cells per well.

For the CRC liver metastasis model, KM12‐SM cells were plated in the upper chamber at a density of 2 × 10^5^ cells per well. Differentiated THP‐1 cells were seeded in the lower insert at a density of 1 × 10^6^ cells in 1.5 mL of RPMI‐1640 with 10% FBS.

### 2.12 Cell Counting Kit 8 (CCK8) Assay

After the 48‐h co‐culture period with CRC cells, differentiated HL‐60 and THP‐1 cells were seeded into 96‐well plates at a density of 1 × 10^4^ cells per well. Different drug treatments were then applied, with DMSO used as a solvent control. At specific time points (6, 12, 24, 36, and 48 h), CCK‐8 reagent (Yeasen, Shanghai, China, Cat: 40203ES80) was added to each well according to the manufacturer's protocol. Plates were incubated for 2 h at 37°C, and absorbance was measured at 450 nm using a Bio‐Tek microplate reader (EL800, Bio‐Tek, USA). Each condition was tested with a sample size of *n* = 6, and experiments were conducted in triplicate to ensure statistical robustness and reproducibility. Results were expressed as mean ± standard deviation (SD).

### 2.13 Western Blot (WB) Assay

HL‐60 and THP‐1 cells were harvested and lysed in RIPA buffer (Beyotime Biotechnology, China, Cat: P0013B), supplemented with protease and phosphatase inhibitors. Protein concentration was determined using the BCA protein assay kit (Abbkine, China, Cat: KTD3001). Equal amounts of protein (30 μg) were separated by 10% SurePAGE (GenScript, China, Cat: M00666) gel electrophoresis and transferred onto PVDF membranes. The membranes were blocked with 5% non‐fat milk in TBST for 1 h at room temperature (25°C–28°C), followed by overnight incubation at 4°C with primary antibodies: anti‐CEACAM19 (1:1000, Thermo Fisher Scientific, USA, Cat: PA5‐115004) and anti‐APP polyclonal antibody (1:1000, Proteintech, USA, Cat: 25524–1‐AP). After washing, the membranes were incubated with a fluorescence‐conjugated secondary antibody (1:5000) for 1 h at room temperature. Protein bands were visualized using the Odyssey Fluorescence Imaging System (LI‐COR Biosciences, Lincoln, NE, USA), and quantification was performed using Image Studio Software (LI‐COR). The relative expression of CEACAM19 and APP was normalized to β‐actin (1:5000, Proteintech, USA, Cat: 66009–1‐Ig).

### 2.14 Cellular Thermal Shift Assay (CETSA)

CETSA experiments were performed according to the general CETSA protocol. Based on the results from the CCK‐8 assay, the maximum concentration of the drug that did not affect cell proliferation was selected for the CETSA experiments. Cells were harvested and resuspended in phosphate‐buffered saline (PBS). Treated samples were aliquoted and heated at different temperatures for 3 min in a PCR plate. Following heat treatment, a protease inhibitor cocktail was added, and cells were lysed by performing three freeze–thaw cycles using liquid nitrogen and a heat block. The cell lysate was centrifuged at 20,000 × *g* for 20 min at 4°C to remove precipitated proteins. The supernatants containing soluble proteins were collected and analyzed by SDS‐PAGE followed by western blot. Band intensities were quantified using ImageJ, and thermal stability curves were generated by normalizing the signals to the lowest temperature point. All experiments were performed in at least three biological replicates.

## 3 Results

### 3.1 Network Pharmacology Analysis of SCP

**FIGURE 1:**
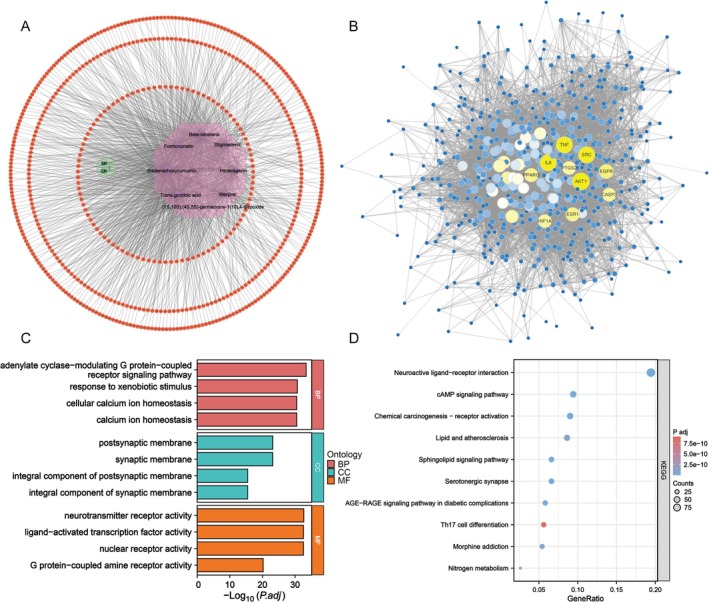
Network pharmacology analysis of (SCP) identifies key active ingredients, potential targets, and mechanistic insights. (A) Identification of 8 primary active ingredients and 566 unique potential targets based on data from the TCMSP, SEA, SwissTargetPrediction, and TargetNet databases. A network of 576 nodes and 1228 interactions was constructed using Cytoscape. Purple nodes represent active ingredients, and orange nodes represent targets. (B) Protein–protein interaction (PPI) network of 566 SCP target proteins, constructed via STRING database with a confidence score of 0.7, showing 557 nodes and 9002 edges. Core hub proteins include TNF, IL6, AKT1, and others involved in inflammatory regulation. (C) Gene ontology (GO) enrichment analysis highlighting biological pathways and molecular functions such as inflammatory response, apoptotic signaling, and receptor activity. (D) Kyoto encyclopedia of genes and genomes (KEGG) pathway analysis of SCP targets, revealing key pathways such as “Neuroactive ligand‐receptor interaction” and “AGE‐RAGE signaling pathway in diabetic complications.”

After searching the TCMSP database, a total of 8 primary active ingredients rich in eligible active substances were identified (Table S1). To enhance the reliability and accuracy of compound identification, these compounds were subsequently validated and supplemented through Q‐Orbitrap high‐resolution mass spectrometry (HRMS) analysis. Detailed parameters of the identified compounds are provided in Figure S1, and Table S2. Next to obtain potential targets associated with these active ingredients, we merged target information from four databases, including TCMSP, SEA, SwissTargetPrediction, and TargetNet. After target normalization and duplicate data elimination using the UniProt database, a total of 566 targets associated with the SCP were determined. The active ingredients and potential targets of SCP were imported into Cytoscape 3.8.0 software to construct a “traditional Chinese medicine‐active ingredients‐potential targets” network containing 576 nodes and 1228 interaction pairs (Figure 1A).

Subsequently, we embarked on a topological analysis of the target network by initially importing all 566 potential targets into the STRING database to construct the PPI network. The resultant data were then imported into Cytoscape software version 3.8.0 for comprehensive network topology analysis and visualization. Our analysis unveiled that the PPI network encompassed 557 targets interconnected through 9002 interactions, possessing an average degree value of 32.32, as depicted in Figure 1B. The degree‐based ranking allowed us to identify 10 core objectives, including TNF, IL6, AKT1, SRC, EGFR, ESR1, EGFR, CASP3, PPARG, and HIF1A (Corcoran and O'Neill 2016). Notably, these molecules play pivotal roles in the intricate network regulating inflammatory responses (Bradley 2008; Dichtl et al. 2021; Lin et al. 2022; Kundu et al. 2011), underscoring their critical involvement in the pathogenesis of inflammatory conditions. SR, in conjunction with CR, has exhibited strong anti‐inflammatory properties in a range of experimental settings (Zou et al. 2021; Jia et al. 2021; Ouyang et al. 2021; Laurindo et al. 2023; Sadeghi et al. 2023). The active constituents within SR and CR are postulated to mediate their anti‐inflammatory actions by modulating inflammatory signaling cascades and inhibiting the secretion of pro‐inflammatory cytokines (Oh et al. 2014; Wu and Tong 2022; Wang et al. 2023). Thus, combining the existing reports and the results of our analysis, the SCP may function pharmacologically mainly through an anti‐inflammatory mechanism.

To gain a deeper understanding of the potential synergistic mechanisms underlying the therapeutic efficacy of SCP in CRC treatment, we performed GO and KEGG functional enrichment analyses on the identified potential targets. The findings are visualized in Figure 1C,D, which, respectively, display histograms and bubble plots illustrating the most statistically significant biological processes and signaling pathways, ranked according to their *p*‐values. These analyses revealed that the therapeutic mechanism of SCP is intricately associated with a diverse array of biological processes and signaling pathways, including G protein‐coupled receptor (GPCR) signaling, calcium ion signaling, nuclear receptor activity, immune regulation, metabolic processes, and other related pathways.

### 3.2 Identification of Cell and Tissue Specificity of SCP by scRNA‐Seq

**FIGURE 2:**
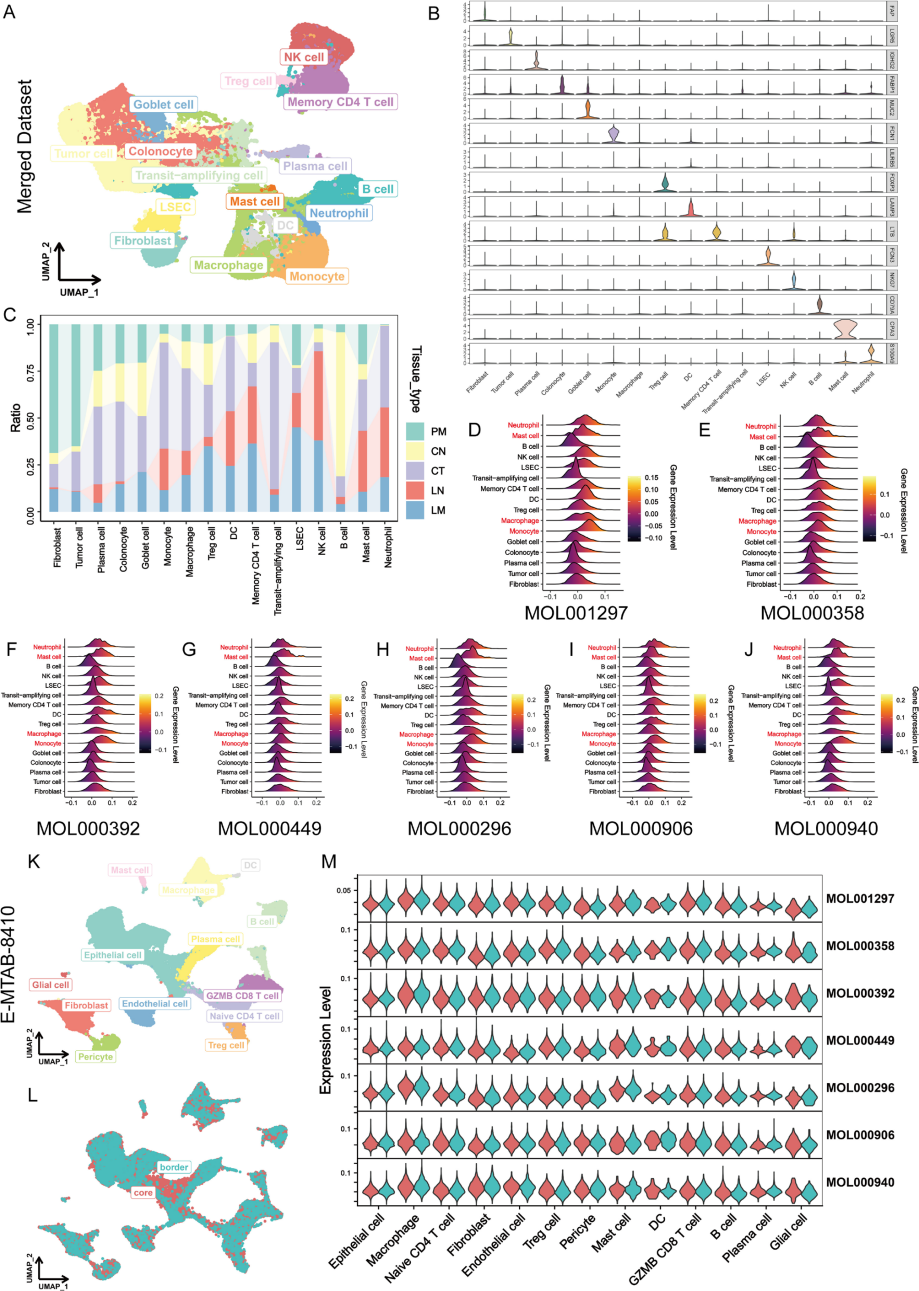
Identification of cell and tissue specificity of SCP by scRNA‐seq. (A) Unsupervised clustering of scRNA‐seq data from 5 primary colorectal tumor samples (CT), 5 liver metastasis samples (LM), 5 peritoneal metastasis samples (PM), 2 normal intestinal samples (CN), and 3 normal liver samples (LN), identifying 16 major cell lineages including tumor cells, fibroblasts, epithelial cells, myeloid cells, and lymphocytes. (B) Violin plot showing the expression levels of well‐known markers for different cell types derived from scRNA‐seq data. Each row corresponds to a specific gene, and the plots illustrate its distribution of expression in the different cell types. (C) Proportional comparison of cell‐type distribution across CT, LM, PM, CN, and LN groups. (D–J) Expression profiles of SCP‐associated targets derived from 7 active ingredients, showing enrichment primarily in myeloid cells such as neutrophils, mast cells, and macrophages. (K) Cell clustering of the E‐MTAB‐8410 dataset reveals detailed immune, stromal, and epithelial compartments in the tumor microenvironment. (L) Spatial distribution of cell types in tumor core versus border regions, indicating core enrichment of tumor cells and neutrophils, and border enrichment of macrophages and fibroblasts. (M) Regional expression of SCP target genes, suggesting spatially distinct pharmacological activity of SCP.

Advancements in single‐cell RNA‐sequencing (scRNA‐seq) technologies have enabled researchers to comprehensively characterize the TME at unprecedented resolution. In this study, we analyzed scRNA‐seq datasets obtained from previously published studies, encompassing 5 primary colorectal tumor samples (CT), 5 liver metastasis samples (LM), 5 peritoneal metastasis samples (PM), 2 normal intestinal samples (CN), and 3 normal liver samples (LN). Following rigorous quality control measures, a total of 99,876 cells were retained for downstream analysis. After correcting for batch effects and performing unsupervised clustering, we identified 16 distinct cell lineages based on canonical marker gene expression profiles and the DISCO annotation tool. These lineages included Fibroblasts, Tumor cells, Plasma cells, Colonocytes, Goblet cells, Monocytes, Macrophages, T regulatory (Treg) cells, Dendritic cells (DCs), Memory CD4+ T cells, Transit‐amplifying cells, Liver sinusoidal endothelial cells (LSECs), Natural killer (NK) cells, B cells, Mast cells, and Neutrophils (Figure 2A). To further verify cell identity and marker specificity, Figure 2B illustrates the expression patterns of representative marker genes across the 16 identified cell lineages. These markers were curated based on canonical gene sets and results from the “FindAllMarkers” function. Notably, the proportion of each cell lineage showed substantial variation across different tissue types, as illustrated in Figure 2C. This analysis underscores the cellular heterogeneity of the TME across primary and metastatic CRC tissues, as well as normal tissues.

Using the “AddModuleScore” function, we mapped the previously derived target expression profiles of the seven active ingredients (excluding MOL000915) onto the scRNA‐seq dataset. As shown in Figure 2D–J, the SCP‐associated targets were predominantly enriched in myeloid lineage cells, including neutrophils, mast cells, and monocytes/macrophages. These findings highlight the selective targeting of SCP on immune cell subsets within the tumor microenvironment and further underscore its potential anti‐inflammatory mechanisms of action.

The therapeutic efficacy of anti‐cancer agents can be profoundly influenced by the spatial location of tumors, as differences in the tumor microenvironment across anatomical sites may significantly impact pharmacodynamics and drug biodistribution (Wu et al. 2018). Additionally, factors such as tumor accessibility and perfusion characteristics at various tumor locations can further affect drug delivery efficiency and therapeutic effectiveness (El‐Sawy et al. 2018; Maestri et al. 2024). Recognizing the critical role of tumor location in treatment outcomes, we incorporated an additional scRNA‐seq dataset, E‐MTAB‐8410, which includes tumor spatial location information, and analyzed the expression profiles of SCP‐related targets (Figure 2K–M).

Interestingly, we observed that the enrichment patterns of targets associated with the seven active ingredients varied based on cell‐type and tumor location. Notably, the specific extracts derived from SR and CR demonstrate the ability to modulate the inflammatory phenotype of macrophages (Zhou et al. 2015). This is evidenced by the down‐regulation of inflammatory cytokine expression and suppression of inflammatory signaling pathway activation (Guimarães et al. 2013). Of particular interest, our findings suggest that SCP exhibits a preferential impact on macrophages located in the tumor border region. These results further emphasize the spatially specific effects of SCP, particularly in modulating macrophage‐associated inflammatory responses in the TME.

### 3.3 SCP Mechanism Correlates With the Inflammatory Phenotype of Neutrophils and Macrophages

**FIGURE 3:**
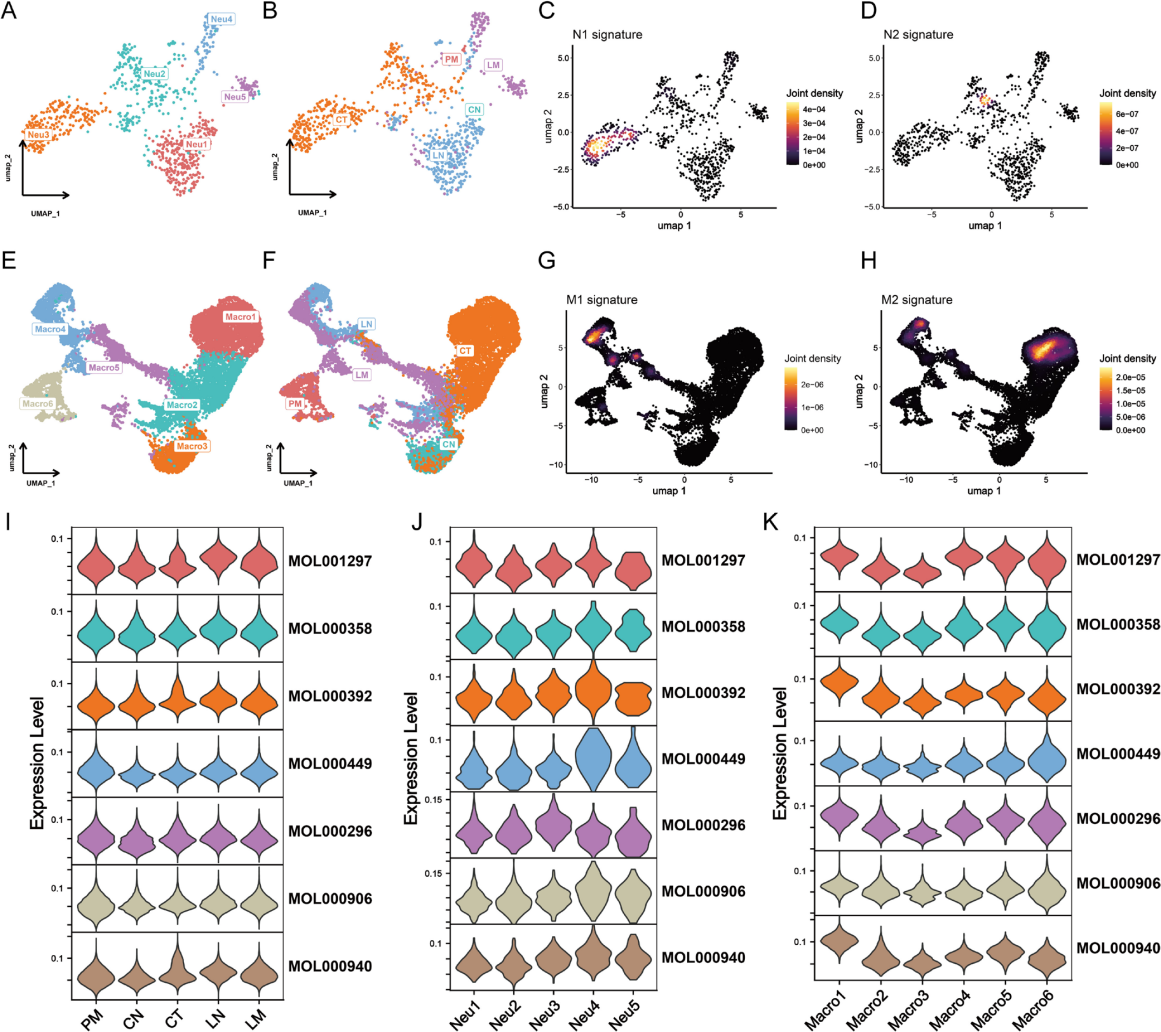
SCP regulates the inflammatory phenotype of neutrophils and macrophages. (A and E) Unsupervised clustering of neutrophils (898 cells) and macrophages (11,809 cells) identified five neutrophil subclusters (Neu1–Neu5) and six macrophage subclusters (Macro1–Macro6). (B and F) The tissue distribution of these subclusters showed that Neu2 and Neu3 were predominantly derived from primary colorectal tumors (CT), while Macro1 was enriched in CT and Macro4 was more abundant in liver metastases (LM) and normal liver (LN). (C and D) Neu2 exhibited a pro‐tumor, anti‐inflammatory (N2‐like) phenotype, whereas Neu3 exhibited a pro‐inflammatory, anti‐tumor (N1‐like) phenotype. (G and H) Macro1, associated with immunosuppressive (M2‐like) properties, was primarily observed in CT, while Macro4, associated with immune activation (M1‐like), was enriched in liver tissues. (I) SCP target genes were preferentially expressed in liver‐associated macrophages, particularly in Macro4. (J and K) SCP‐associated targets are predominantly enriched in neutrophils with an N1‐like phenotype, while no significant differences in expression are found between M1‐ and M2‐like macrophages.

Given that SCP demonstrated a potential modulatory effect on myeloid cells, we further investigated its impact on neutrophils and macrophages. After performing unsupervised cell clustering, we identified five distinct neutrophil subclusters (Neu1‐5) from a total of 898 neutrophils and six macrophage subclusters (Macro1‐6) from 11,809 macrophages (Figure 3A,E). The tissue origins of neutrophils and macrophages are depicted in Figure 3B,F, respectively. Notably, Neu2 and Neu3 represented the predominant neutrophil subtypes in colorectal tumor (CT) samples, with Neu2 exhibiting an N2‐like phenotype and Neu3 exhibiting an N1‐like phenotype (Figure 3C,D). Among macrophages, Macro1 was the most abundant type in CT, exhibiting M2‐like features, whereas Macro4 was the dominant macrophage subtype in liver samples (LM and LN), displaying M1‐like characteristics (Figure 3G,H). Subsequently, we analyzed the expression profiles of SCP‐associated targets in these cell populations and found a significant enrichment of these targets in liver samples (Figure 3I). To further investigate, we compared the distribution of SCP‐related targets across the different neutrophil and macrophage subpopulations (Figure 3J,K). Our results revealed a preferential involvement of SCP in neutrophils with the N1 phenotype, while no significant differences were observed between M1‐ and M2‐like macrophages. These findings suggest that SCP may selectively modulate neutrophils exhibiting an N1‐like phenotype, potentially influencing the inflammatory microenvironment in the tumor and surrounding tissues.

### 3.4 SCP Predominantly Target Neutrophil‐Dominated Spatial Domains

TCM in cancer treatment employs a multi‐target, multi‐cellular, and multi‐signaling approach (Yang, Zhang, et al. 2021). In contrast to conventional monotherapy, TCM utilizes complex formulations that address cancer from multiple angles, acknowledging its inherent heterogeneity (Wang, Li, et al. 2024). By targeting a broad spectrum of molecular pathways, TCM aims not only to inhibit tumor growth but also to alleviate symptoms and modulate the immune response (Fu et al. 2024). Given that SCP exhibits specificity for cells in distinct spatial locations, we hypothesized that SCP may selectively target specific spatial domains or cellular collections within the tumor microenvironment. This spatial specificity could potentially enhance the therapeutic efficacy of SCP by modulating key cellular interactions in a more localized and context‐dependent manner.

**FIGURE 4:**
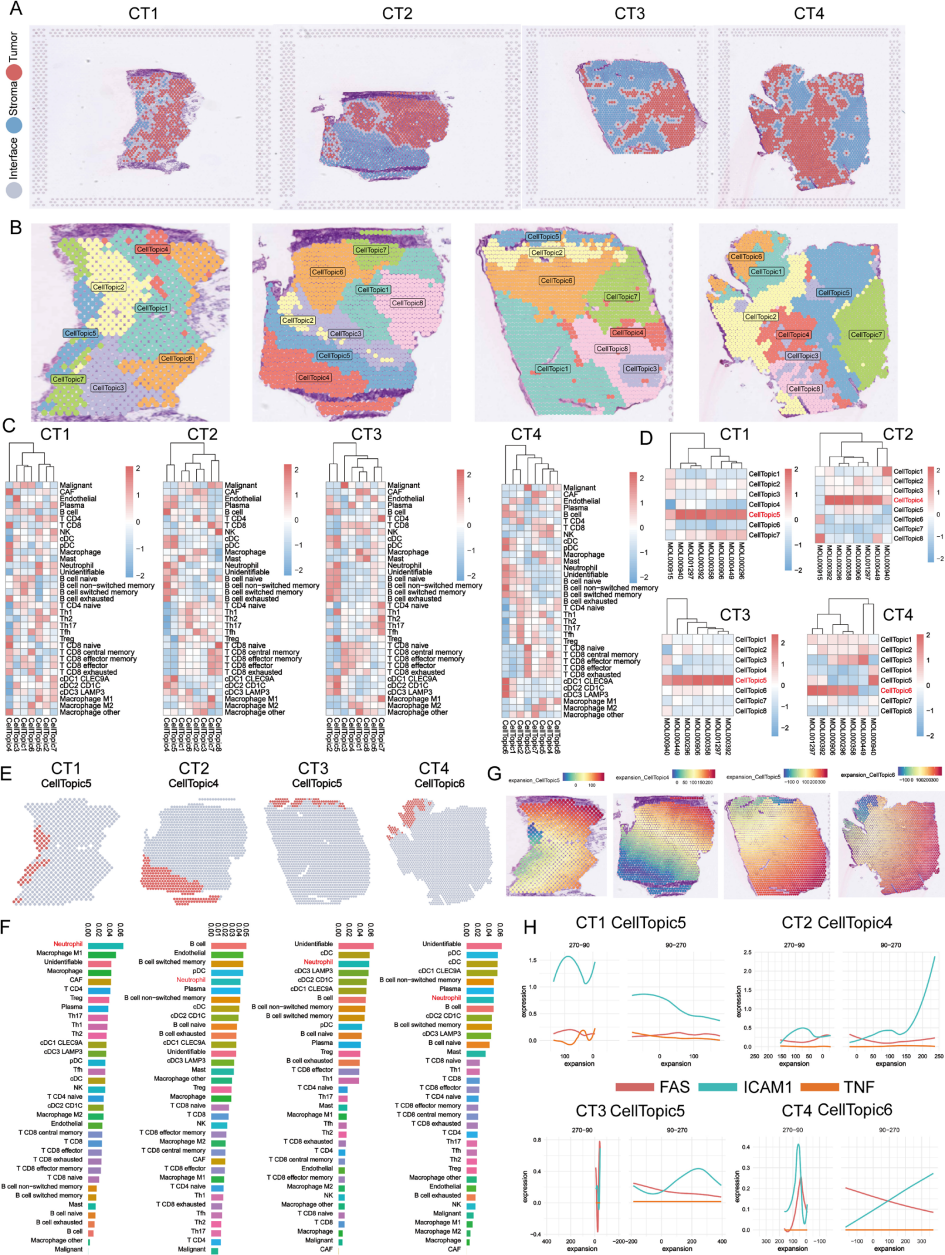
SCP targets neutrophil‐dominated spatial domains in CRC tumor samples. (A) Spatial transcriptomics data from four primary CRC tumor samples (CT1–CT4) were analyzed to identify spatial gene expression patterns. Tumor regions (red), stromal regions (blue), and tumor‐stroma interface regions (gray) were defined based on spatial expression profiles. (B) The low‐resolution nature of Visium data was addressed using an integrated framework combining the “BayesSpace” spatial clustering method and the “SPCET” cell proportion estimation method. The integration through “SpaTopic” allowed the categorization of spatial spots into seven topics in CT1 and eight topics in CT2–CT4, reflecting distinct regions within the tumor microenvironment (see also Figure S2). (C) Heatmaps showing the cellular composition of identified spatial domains, with unique mixtures of immune, stromal, and tumor cell types across the different regions, highlighting the tumor's cellular heterogeneity. (D) SCP‐related targets were mapped onto the spatial topics, revealing preferential targeting of specific domains. SCP predominantly targeted Topic5 in CT1, Topic4 in CT2, Topic5 in CT3, and Topic6 in CT4, suggesting selective interaction with regions enriched in specific cell types or pathways (see also Figure S3). (E and F) Analysis of cellular composition within SCP‐targeted domains demonstrated that neutrophils were consistently the dominant cell type, particularly in regions enriched with N1‐like neutrophils. These regions, associated with anti‐tumor activity, indicate SCP's potential to modulate neutrophil‐driven immune responses (see also Figure S4). (G and H) Expression gradients of N1‐like neutrophil marker genes (FAS, ICAM1, and TNF) were analyzed in SCP‐targeted domains. Expression levels of these markers decreased as the distance from SCP‐targeted domains increased (H), indicating SCP's selective modulation of neutrophil activity in specific regions of the tumor microenvironment.

To validate this hypothesis, we retrieved data from the CROST portal for four primary CRC tumor samples (CT1‐CT4). Using the gene expression signature of each sample, we divided the spatial organization into distinct regions, including the tumor, stroma, and interface areas (Figure 4A). Given the low‐resolution nature of Visium technology (where each spot contains multiple cells), we applied the “BayesSpace” method for spatial clustering of the CT samples and used the “SPCET” method to identify cell proportions. These two approaches were integrated using the “SpaTopic” method to categorize the spots into several cellular topics, or spatial domains.

As depicted in Figures 4B and S2, seven distinct topics were identified in CT1, while CT2–CT4 each revealed eight topics. These cellular topics effectively represent specific areas within the tumor samples. Interestingly, the cellular composition of these domains exhibited considerable variation, as shown in Figure 4C. Subsequently, we projected the potential targets of SCP onto the spatial organization to identify the specific domains that SCP may target. Notably, SCP exhibited distinct targeting patterns across the samples, with specific domains targeted in different tumor regions: Topic 5 in CT1, Topic 4 in CT2, Topic 5 in CT3, and Topic 6 in CT4 (Figures 4D and S3). Upon further dissection of the cellular composition within these spatial domains, we observed that neutrophils were consistently a dominant cellular component (Figures 4E,F and S4). This finding further corroborates our previous conclusion that neutrophils are likely one of the primary targets of SCP.

Additionally, using the “Expansion” function, we analyzed the expression gradient of N1‐like marker genes (FAS, ICAM1, TNF) associated with the domains targeted by SCP. As expected, the expression of N1 markers tended to decrease with increasing distance from the SCP‐targeted domains (Figure 4G,H). These results suggest that SCP may exert its therapeutic effects by selectively targeting domains predominantly composed of N1‐like neutrophils, thereby modulating the tumor microenvironment in a spatially specific manner.

### 3.5 CEACAM Signaling May Be a Potential Downstream Target of SCP

**FIGURE 5:**
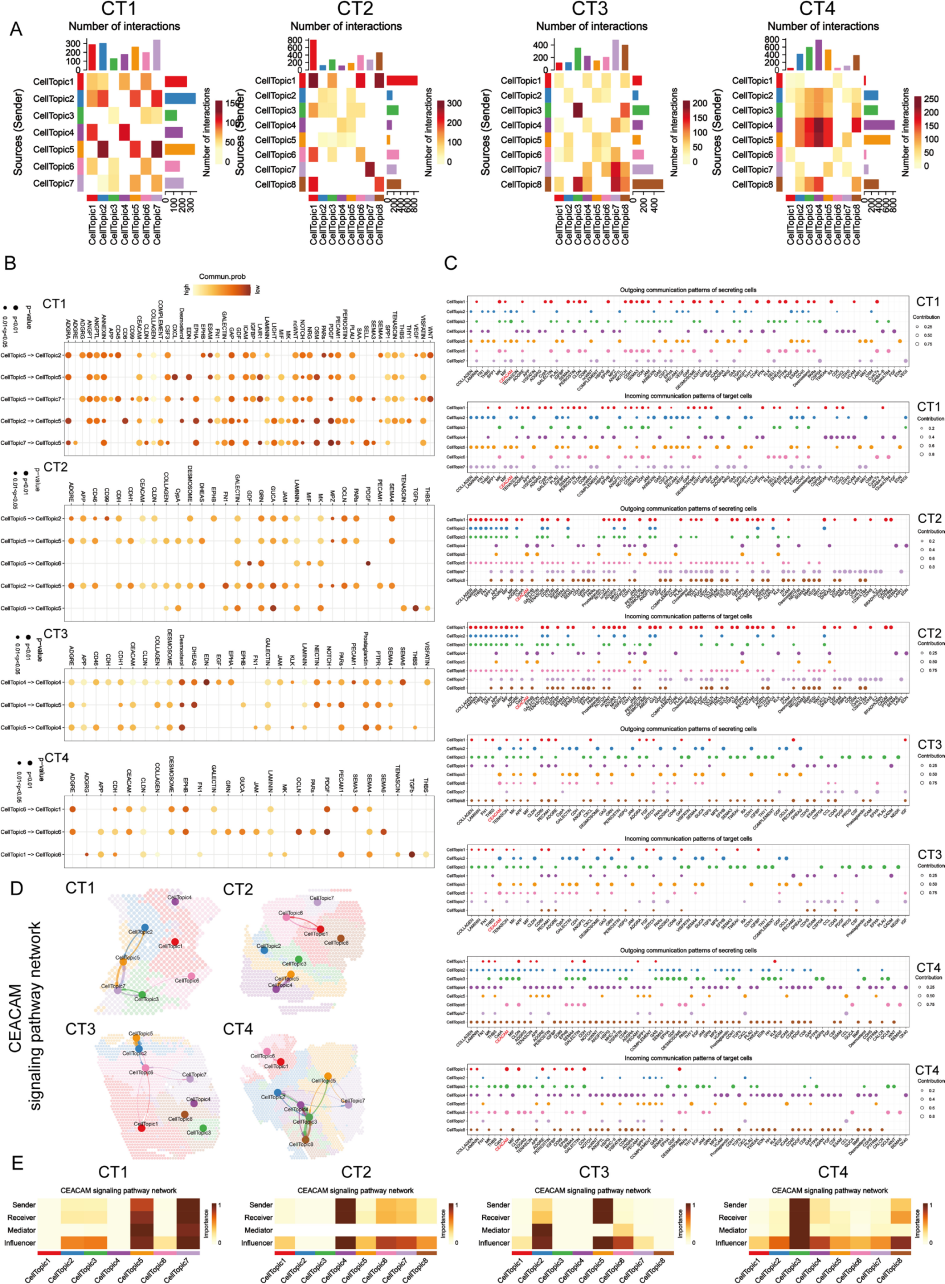
SCP‐targeted spatial domains regulate TME communication via CEACAM signaling. (A) The spatial organization of cellular communication in tumor samples was analyzed using the “CellChat” method. Active signaling was identified within SCP‐targeted domains (Topic 5 in CT1, Topic 4 in CT2, Topic 5 in CT3, and Topic 6 in CT4), as well as between these domains and adjacent regions. Warmer colors and thicker lines indicate stronger communication activity, with the bar graph quantifying signaling intensity in each region. (B) CEACAM signaling plays a central role in the communication network. The circle size represents the signaling strength of each spatial domain, with larger circles reflecting higher signaling activity. Bar color intensity indicates signaling levels, where lighter bars correspond to stronger activity. (C) Domain‐specific communication analysis revealed that CEACAM signaling is a crucial component of both incoming and outgoing signaling processes within SCP‐targeted regions, especially in neutrophil‐rich domains. The circle size represents the intensity of communication, with color denoting different cellular topics (spatial domains). (D) CEACAM signaling flow was visualized to show directional communication within the TME. SCP‐targeted domains consistently exhibit high CEACAM signal transmission, particularly in neutrophil‐enriched areas, suggesting SCP's role in modulating TME communication. (E) Network centrality analysis identified SCP‐targeted domains as key hubs in the CEACAM signaling network within the TME. These domains are major senders and regulators of signaling, with warm colors highlighting their involvement in CEACAM transmission. The scale on the right shows interaction importance, where higher values (closer to 1) indicate stronger influence.

Since different spatial domains within the TME may represent functionally distinct zones that form consistent spatial organizations, exploring the communication between these domains is crucial for understanding how SCP regulates the TME, both locally and globally. To achieve this, we inferred the communication probabilities among and within these domains using the “CellChat” method. Our analysis revealed that the SCP‐targeted domains—Topic 5 in CT1, Topic 4 in CT2, Topic 5 in CT3, and Topic 6 in CT4—not only exhibited internal communication within their respective domains but also showed potential interactions with other domains (Figure 5A).

Further examination suggested that these SCP‐targeted domains play a pivotal role in mediating signal crosstalk within the TME, utilizing a wide range of signaling pathways (Figure 5B). Among the signals involved, CEACAM signaling emerged as a critical mediator, particularly in the differentiation trajectory and maturation of tumor‐associated neutrophils (TANs) (Rayes et al. 2020). This signaling influences their polarization and functional orientation (Skubitz and Skubitz 2008), which is particularly relevant given that the SCP‐targeted domains are neutrophil‐dominated. Consequently, we focused on CEACAM signaling in the context of SCP's effects.

Using pattern recognition methods, we identified domain‐specific communication patterns and found that CEACAM signaling plays a central role in both incoming and outgoing signaling within the SCP‐targeted domains (Figure 5C). The spatial flow of CEACAM signaling is illustrated in Figure 5D. To further elucidate the importance of CEACAM signaling in the communication networks associated with SCP‐targeted domains, we calculated the network centrality scores for each domain. Remarkably, the SCP‐targeted domains consistently acted as the primary senders and key influencers of CEACAM signaling across all four CT samples (Figure 5E). This result suggests a significant link between CEACAM signaling and SCP intervention, highlighting the potential for SCP to modulate the polarization and functional orientation of neutrophils through CEACAM‐mediated signaling pathways.

### 3.6 SCP Down‐Regulate the Expression Level of CEACAM19 in CT

**FIGURE 6:**
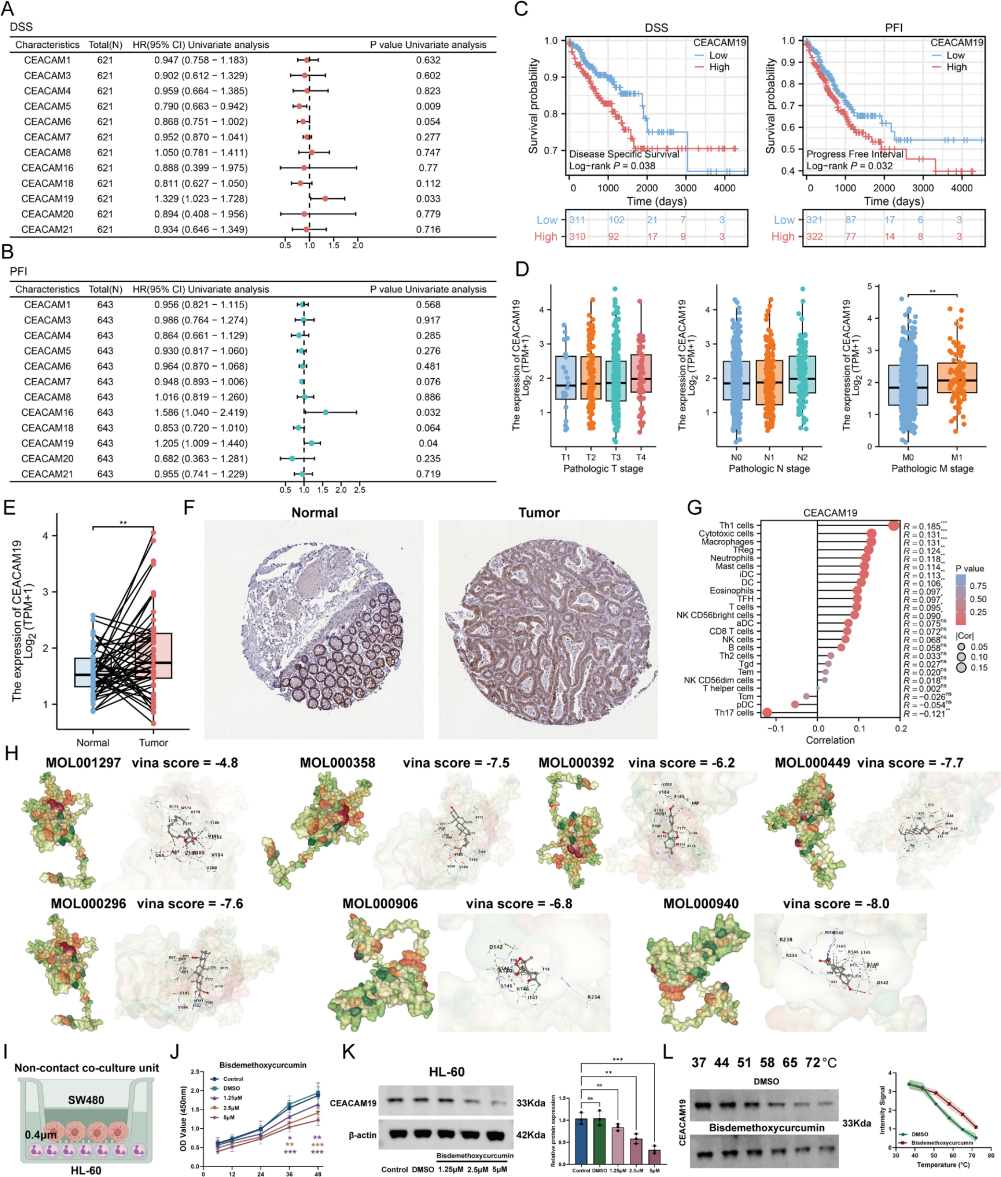
SCP suppresses colorectal cancer progression partly through down‐regulation of CEACAM19. (A and B) Univariate Cox regression analyses showing that high *CEACAM19* expression is significantly associated with poor prognosis in colorectal cancer. Elevated CEACAM19 expression correlates with shorter disease‐specific survival (DSS; HR = 1.329, *p* = 0.033) and progression‐free interval (PFI; HR = 1.205, *p* = 0.04). (C) Kaplan–Meier survival curves further supported the detrimental role of *CEACAM19*, with high‐expression groups exhibiting significantly lower DSS (*p* = 0.038) and PFI (*p* = 0.032) compared to low‐expression groups. (D) Clinical stage‐based expression patterns of CEACAM19 (log_2_TPM + 1), with notably higher expression in M1‐stage patients (*p* < 0.01), suggesting a potential role in metastasis. (E) Boxplot and paired scatter plots show elevated CEACAM19 expression in tumor tissues versus matched normal tissues. (F) Immunohistochemistry (IHC) staining obtained from the Human Protein Atlas (HPA) database confirms minimal CEACAM19 expression in normal colorectal tissue and marked upregulation in tumor samples. (G) Immune cell infiltration correlation indicates positive associations between CEACAM19 expression and multiple immune cell types (Th1 cells, cytotoxic cells, macrophages, Tregs, neutrophils, mast cells). Circle size reflects correlation strength; color intensity denotes statistical significance. (H) Molecular docking simulations identify potential CEACAM19‐binding compounds. Surface renderings illustrate binding affinities, with green/red regions indicating favorable interactions. MOL000940 exhibits the strongest binding (Vina score = −8.0), suggesting its potential as a CEACAM19 inhibitor for therapeutic development. (I) Schematic of the non‐contact co‐culture model using SW480 colorectal cancer cells (upper chamber) and DMSO‐differentiated HL‐60 neutrophil‐like cells (lower chamber) in Transwell inserts (0.4 μm). (J) CCK‐8 assay measuring cell viability of HL‐60 neutrophil‐like cells after 48 h co‐culture with SW480 cells, followed by treatment with Bisdemethoxycurcumin (0, 1.25, 2.5, 5 μM) or vehicle control (DMSO) for 6, 12, 24, 36, and 48 h. (ANOVA with Tukey's post hoc test to compare multiple concentrations and time points) Concentrations showing no significant impact on cell viability (*p* > 0.05) were selected for subsequent experiments. (*n* = 6, mean ± SD, *p < 0.05, **p < 0.01, ***p < 0.001). (K) Western blot analysis of CEACAM19 and β‐actin (internal reference) in HL‐60 neutrophil‐like cells from SW480/HL‐60 co‐cultures treated with Bisdemethoxycurcumin or DMSO for 24 h. (ANOVA, *n* = 3, mean ± SD, ns = not significant, **p < 0.01, ***p < 0.001) (L) Cellular thermal shift assay (CETSA) of CEACAM19 in HL‐60 cells from co‐cultures treated with 5 M Bisdemethoxycurcumin or DMSO for 24 h.

We performed univariate Cox regression analysis on the 12 CEACAM family members to identify potential prognostic factors and found that only *CEACAM19* was a significant detrimental factor for both disease‐specific survival (DSS) and progression‐free interval (PFI) (Figure 6A,B). The Kaplan–Meier survival curves further demonstrated that patients with high *CEACAM19* expression had significantly lower DSS (*p* = 0.038) and PFI (*p* = 0.032) rates compared to those with low CEACAM19 expression (Figure 6C).

Additionally, we examined *CEACAM19* expression across different clinical stages of CRC in the TCGA‐CRC cohort and observed a positive correlation between *CEACAM19* expression and the M stage (Figure 6D,P < 0.01). To further validate these findings, we analyzed paired tissue samples from TCGA and IHC images from the Human Protein Atlas (HPA), which revealed that CEACAM19 expression was significantly higher in cancer tissues compared to normal controls (Figure 6E,F). Furthermore, *CEACAM19* expression showed positive correlations with several immune cell types, including Th1 cells, cytotoxic cells, macrophages, Tregs, neutrophils, and mast cells (Figure 6G). These findings collectively suggest that CEACAM19 may play a key role in the progression of CRC.

Given the potential association between SCP and CEACAM signaling, we next performed molecular docking of the seven active components in SCP with the identified prognostic risk factor CEACAM19. As anticipated, these active ingredients demonstrated favorable docking interactions with CEACAM19, with corresponding Vina scores of −4.8, −7.5, −6.2, −7.7, −7.6, −6.8, and −8.0, respectively (Figure 6H). These results suggest that the therapeutic effect of SCP in suppressing tumor progression may, at least in part, be mediated through modulation of CEACAM19 signaling. To experimentally validate these computational predictions, we employed a non‐contact co‐culture model of SW480 cells (derived from a primary colorectal adenocarcinoma) and HL‐60‐derived neutrophil‐like cells (Figure 6I). CCK‐8 assays showed that Bisdemethoxycurcumin at concentrations ≤ 5 μM had no significant cytotoxicity on neutrophil‐like cells within 24 h, and thus these doses were used for subsequent experiments (Figure 6J). Western blot analysis revealed that Bisdemethoxycurcumin reduced CEACAM19 expression in a dose‐dependent manner in HL‐60 cells (Figure 6K). CETSA further showed that Bisdemethoxycurcumin increased the thermal stability of CEACAM19, indicating a direct interaction between the compound and the protein (Figure 6L).

### 3.7 SCP Predominantly Target Macrophage‐Dominated Spatial Domains in LM

**FIGURE 7:**
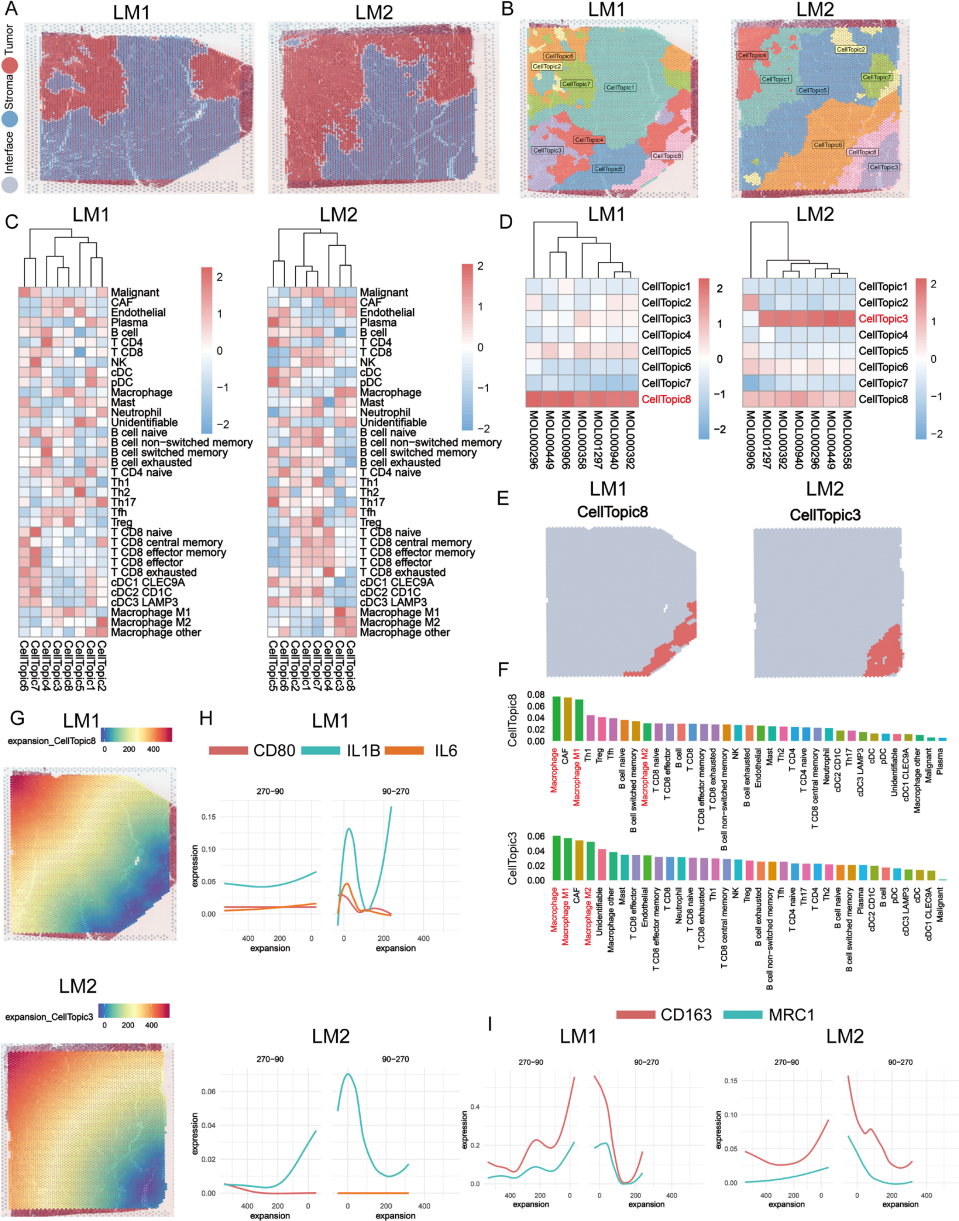
SCP primarily targets macrophage‐dominated spatial domains in liver metastases (LM). (A) Spatial transcriptomics (ST) analysis of two liver metastasis samples (LM1 and LM2) identifies distinct tissue regions based on gene expression profiles. The sections are categorized into tumor regions (red), stromal regions (blue), and tumor‐stroma interface regions (gray). (B) Cellular annotation of LM tissues reveals eight distinct cell topics across LM1 and LM2 samples, color‐coded for each cluster with similar gene expression profiles. The legend specifies each cell topic. (C) Heatmap of cellular composition shows the relative abundance of cell types within each cell topic across both LM samples. Red indicates higher cell abundance, while blue indicates lower abundance (also see Figure S5). (D) SCP‐associated spatial domains were identified by mapping SCP‐related target expression profiles onto ST data. In LM1, CellTopic8, and in LM2, CellTopic3, were strongly associated with SCP, suggesting SCP's preferential targeting of specific regions within LM tissues (also see Figure S6). Darker shades indicate stronger SCP‐related expression. (E) Spatial distribution of SCP‐associated domains shows that CellTopic8 in LM1 and CellTopic3 in LM2 are enriched in SCP‐related activity (also see Figure S7). (F) Cellular composition analysis of SCP‐associated domains in LM1 (CellTopic8) and LM2 (CellTopic3) reveals a high abundance of macrophages, including both M1 and M2 subtypes. These macrophage‐dominated regions (marked in red font) also contain significant amounts of fibroblasts (CAF), highlighted in the bar charts, indicating that SCP targets immune‐rich and fibroblast‐enriched regions within the tumor microenvironment. (G–I) Macrophage functional analysis shows the expression of M1‐like (pro‐inflammatory, anti‐tumor) markers (CD80, IL1B, IL6) in panels (G, H), and M2‐like (pro‐tumor, anti‐inflammatory) markers (CD163, MRC1) in panel (I). The expression of these markers decreases as the distance from SCP‐associated domains increases, suggesting SCP's role in targeting macrophages, potentially influencing macrophage polarization and functional states, thus modulating tumor progression and immune regulation.

The scRNA‐seq study suggests that SCP might have a role in inhibiting LM; to further explore this possibility, we searched for SCP‐related spatial domains in LM tissues. For this, we utilized spatial transcriptomics (ST) data from two LM samples (LM1‐LM2) collected from a previous study. Based on gene expression patterns, the LM tissues were categorized into distinct regions, including normal, interface, and stroma (Figure 7A). Additionally, similar to CT, LM tissues were annotated into different cell topics (8 topics in LM1 and LM2, respectively) (Figures 7B and S5). These topics were differentiated based on their distinct cellular compositions (Figure 7C).

We then mapped the potential targets of the seven active components in SCP onto the ST data and identified spatial domains associated with SCP in LM1 (CellTopic8) and LM2 (CellTopic3) (Figures 7D and S6). Notably, these SCP‐associated domains were predominantly composed of macrophages and were characterized by high levels of fibroblasts (Figures 7E,F and S7). Given the scRNA‐seq findings suggesting that SCP may regulate both M1‐like and M2‐like macrophages, we further examined the gene expression gradient of SCP‐related domains.

As shown in Figure 7G–I, the levels of both M1 and M2 macrophage markers decreased as the distance from the SCP‐associated domains increased. This observation indicates that SCP may primarily target macrophage‐dominated spatial domains in LM, suggesting a potential mechanism through which SCP influences the tumor microenvironment and macrophage activity in LM tissues.

### 3.8 SCP Down‐Regulate the Expression Level of APP in LM

**FIGURE 8:**
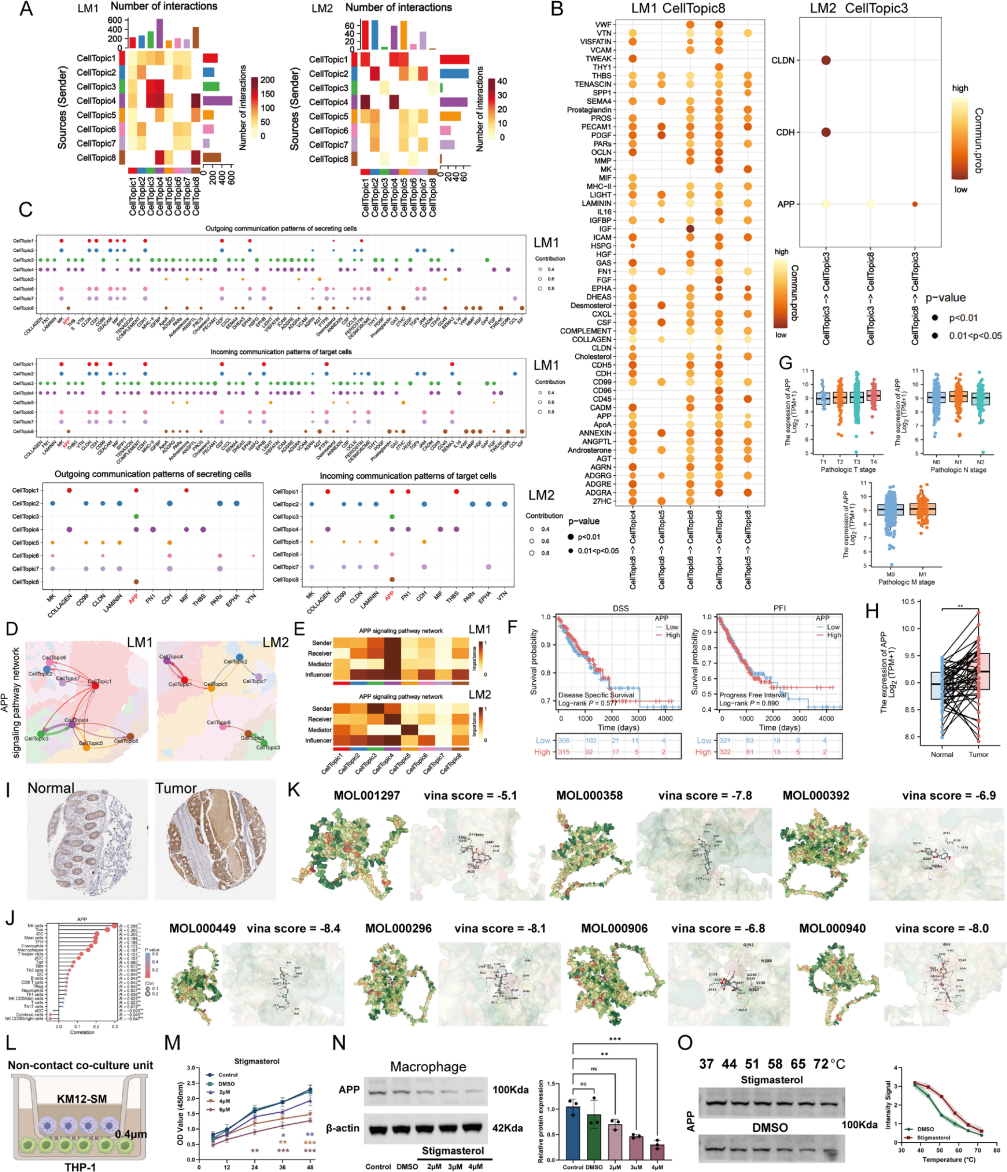
SCP suppresses APP signaling in liver metastases (LM). (A) Domain‐domain communication analysis in LM1 and LM2 reveals active interactions between SCP‐related domains and neighboring domains. The color gradient indicates interaction strength, with dark red representing strong interactions and yellow representing weaker ones. CellTopic1 and CellTopic3 show the most active communication (dark red), while interactions in CellTopic5 and CellTopic8 are weaker (light yellow). Bar graphs summarize the total number of interactions for each cell topic. (B) Communication probabilities between cell topics in LM1 (CellTopic8) and LM2 (CellTopic3) are represented by circle size and color. Larger circles indicate stronger communication, and smaller circles reflect weaker interactions. Darker orange circles represent lower communication probabilities, while lighter orange circles correspond to higher probabilities. APP signaling is the only SCP‐related signaling observed in both LM samples. (C) Domain‐specific communication patterns highlight the central role of APP signaling in SCP‐related domains. APP signaling is prominent in both intra‐ and inter‐domain communication within SCP‐targeted regions, emphasizing its key role in SCP's mechanism of action. Circle color indicates different cell topics, and circle size reflects the contribution of communication strength. (D) APP signaling network in LM1 (left) and LM2 (right) shows interactions between cell topics. Circle colors represent different cell topics. The line thickness indicates interaction strength, with thicker lines representing stronger interactions. (E) APP signaling pathway network in LM1 (top) and LM2 (bottom) depicts interaction dynamics between sender, receiver, mediator, and influencer cell topics. Heatmaps show the importance of interactions, with dark red indicating high importance and light yellow indicating low importance. The scale on the right indicates the importance of interactions. (F) Survival analysis of APP expression in LM tumors. Kaplan–Meier curves for disease‐specific survival (DSS) (left) and progression‐free interval (PFI) (right) show survival probabilities for low (blue) and high (red) APP expression. Log‐rank *p*‐values: DSS (*p* = 0.57), PFI (*p* = 0.89). (G) *APP* expression across clinical stages is shown by boxplots of log_2_(TPM + 1) for T, N, and M stages. No significant association between APP expression and clinical stages was found. (H and I) APP expression in CRC tissues is upregulated compared to normal controls, supported by both mRNA (TCGA dataset) and protein (IHC images from public databases) expression data. (J) Correlation of APP expression with immune cell populations shows a positive correlation with NK cells, T cells, DCs, mast cells, and macrophages. Larger circles indicate stronger correlations (higher R values), while smaller circles reflect weaker correlations. The color gradient indicates statistical significance, with red indicating strong correlations (low *p*‐values) and blue indicating weak correlations (high *p*‐values). NK cells, Tcm, and iDC show the strongest correlations. (K) Molecular docking of potential APP‐binding compounds shows the vina score for each molecule. The structures are shown on the left, and the binding poses relative to APP are shown on the right. Lower vina scores indicate stronger binding, with MOL000449 (−8.4) exhibiting the strongest interactions. (L) Schematic of non‐contact co‐culture of KM12‐SM cells (upper chamber) with PMA‐differentiated THP‐1 macrophages (lower chamber) in transwell inserts (0.4 μm). (M) CCK‐8 assay of THP‐1 macrophages viability after 48‐h co‐culture with KM12‐SM cells and treatment with stigmasterol (2, 4, 6 μM) or DMSO for 6, 12, 24, 36, and 48 h. The 4 μM stigmasterol treatment for 24 h (*p* > 0.05) was selected for downstream assays as non‐cytotoxic. (N) Western blot of APP and β‐actin in THP‐1 macrophages from co‐cultures treated with stigmasterol or DMSO for 24 h (ANOVA, *n* = 3, mean ± SD, ns = not significant, *p < 0.05, **p < 0.01, ***p < 0.001). (O) CETSA of APP in THP‐1 macrophages treated with 4 μM stigmasterol or DMSO for 24 h.

We also investigated the domain‐domain interactions in the ST of LM tumors, which revealed communication between SCP‐related domains and neighboring regions (Figure 8A). Further analysis showed that the SCP‐associated domains communicate with other domains through multiple signaling pathways; however, only APP signaling was observed in both LM1 and LM2 (Figure 8B). We examined the specific communication patterns of each domain and found that APP signaling plays a crucial role in the interaction between SCP‐related domains (Figure 8C). Figure 8D illustrates the spatial localization of APP signaling within the LM tumor. Notably, the SCP‐related domain emerged as the main influencer of APP signaling (Figure 8E).

To explore the clinical relevance of APP in CRC, we assessed its correlation with several clinical parameters. Although APP did not show significant associations with many common clinical features (Figure 8F,G), its expression was notably upregulated in cancer tissues, as confirmed by both mRNA and protein data (Figure 8H,I). Since APP signaling is closely linked with macrophages in the spatial organization and has been shown to influence macrophage phenotypes—affecting their functional characteristics and responses to various stimuli—we calculated the correlation between APP transcript levels and immune cell infiltration (Liu et al. 2024; Chen et al. 2024). As shown in Figure 8J, APP expression was positively correlated with the abundance of several immune cells, including NK cells, T cells, dendritic cells (DCs), mast cells, and macrophages.

We then examined whether SCP interferes with APP signaling. Molecular docking results suggest that the seven main active ingredients in SCP may interact with APP (Figure 8K). To investigate the impact of SCP on APP signaling in the LM, we used KM12‐SM, a CRC cell line derived from liver metastasis, co‐cultured with PMA‐differentiated THP‐1 macrophages in a non‐contact Transwell system (Figure 8L).

CCK‐8 assays confirmed that 4 μM Stigmasterol for 24 h was non‐cytotoxic to macrophages; this dose was used in follow‐up experiments (Figure 8M). Western blot showed that Stigmasterol significantly reduced APP protein levels in THP‐1 macrophages (Figure 8N). Moreover, CETSA demonstrated increased thermal stability of APP upon treatment (Figure 8O), supporting a direct interaction between Stigmasterol and APP. Together, these findings indicate that Stigmasterol, as a representative active component of SCP, may directly bind APP and suppress its expression in macrophages, providing functional evidence for SCP's modulation of the APP signaling in CRC liver metastases.

## 4 Discussion

CRC poses a substantial global health burden, driven by a complex interplay of environmental, genetic, and lifestyle factors. Its incidence is particularly high in developed countries due to Westernized diets, sedentary lifestyles, aging populations, and familial predispositions. TCM, with its holistic emphasis on maintaining physiological balance and harmony, presents a complementary therapeutic strategy for CRC management (Wang et al. 2020). By integrating herbal formulations, acupuncture, moxibustion, and dietary therapy tailored to the individual's constitutional type and disease stage, TCM aims to alleviate symptoms, enhance the quality of life, potentially augment the efficacy of conventional treatments, and address the multifactorial nature of CRC (Jiang et al. 2023; Zhao et al. 2021; Kong et al. 2020). This study offers a preliminary perspective on how traditional Chinese medicine such as SCP may exert domain‐specific regulatory effects within the tumor microenvironment, suggesting that spatially localized analysis frameworks can aid in uncovering new mechanisms and potential therapeutic targets relevant to tumor progression.

The herbal pair SR and CR, particularly their extracts and active compounds, have attracted significant attention for their potent anti‐inflammatory effects, particularly in myeloid cells like neutrophils and macrophages (Jia et al. 2021; Karimian et al. 2017; Memarzia et al. 2021). Active constituents such as sparganine and curcuminoids have been shown to inhibit neutrophil activation and migration, thereby reducing the release of pro‐inflammatory mediators. Additionally, these compounds promote macrophage polarization from the pro‐inflammatory M1 phenotype to the anti‐inflammatory M2 phenotype, facilitating inflammation resolution and tissue repair (Zou et al. 2021; Franck et al. 2008). Additionally, these compounds promote macrophage polarization from the pro‐inflammatory M1 phenotype to the anti‐inflammatory phenotype, facilitating inflammation resolution and tissue repair (Abdollahi et al. 2023; Nasra et al. 2023). Mechanistically, SR and CR act through the inhibition of NF‐κB and MAPK signaling pathways, two key regulators of pro‐inflammatory cytokine and chemokine production (Cai et al. 2022). These findings highlight the therapeutic potential of SR and CR in modulating myeloid cell‐mediated inflammation and addressing inflammatory diseases.

In this study, we integrated spatial genomics, single‐cell genomics, and network pharmacology to investigate the therapeutic mechanisms of SR and CR in modulating myeloid‐dominated spatial domains within TME. Our findings revealed that the TME consists of interconnected spatial compartments that collectively drive tumor progression. Notably, SCP demonstrated distinct cell domain‐specific effects on primary and metastatic CRC lesions, suggesting that its therapeutic effects may arise from targeting specific spatial domains within the TME. This provides a novel perspective on how spatially localized interventions can enhance anti‐tumor efficacy.

The formation of primary CRC lesions is typically associated with chronic inflammation driven by local neutrophil infiltration (Zhang, Zhang, Zhang, et al. 2023). Our findings demonstrated that SCP primarily acts on neutrophil‐enriched cell domains within primary lesions. This observation aligns with the pathological characteristics of CRC primary tumors and suggests that SCP may inhibit tumor growth and progression by modulating neutrophil activity and restoring the dynamic balance of the local inflammatory microenvironment. Moreover, we identified significant enrichment of CEACAM signaling in primary lesions, which is closely associated with neutrophil adhesion and activation (Skubitz 2024). SCP may influence CEACAM‐related signaling pathways, which are associated with neutrophil activity, and thereby potentially modulate early inflammatory responses within the TME. In CRC liver metastases (LM), SCP primarily acts on macrophage‐enriched cell domains. Additionally, in LM1 (CellTopic8) and LM2 (CellTopic3), cancer‐associated fibroblasts (CAFs) were identified as critical cellular components alongside macrophages. The high proportion of CAFs is closely associated with the pronounced fibrosis and immunosuppressive microenvironment observed in metastatic lesions (Sathe et al. 2023). This heterogeneity is further reflected by the significant enrichment of APP signaling in macrophages within liver metastases. APP signaling has been implicated in macrophage polarization (Liu et al. 2024) and extracellular matrix remodeling (Beyreuther et al. 1996), both of which are crucial for metastatic progression. By focusing on dominant cell domains, such as macrophages, and exploring their associated APP signaling networks, this analysis framework may help reveal how SCP components could influence cell–cell interactions and fibrosis‐related signaling in metastatic lesions. These results suggest that SCP may modulate the functions of dominant cellular domains and their associated signaling pathways, offering insights into how domain‐specific interventions could influence tumor microenvironmental dynamics.

Based on these results, SCP exhibits unique advantages in CRC therapy within the framework of TCM. Unlike conventional anti‐cancer strategies that often emphasize single‐component and single‐targeted mechanisms, SCP operates in a manner consistent with the TCM principle of “treatment based on syndrome differentiation.” This study proposes a preliminary framework suggesting that SCP may differentially affect key cellular domains and their microenvironments in primary and metastatic lesions. For instance, in primary lesions, SCP primarily regulates chronic inflammation within the microenvironment, possibly through CEACAM‐related signaling pathways. In patients with metastatic lesions, optimizing the dosage of SCP within the compound formulation can safely alleviate fibrotic and immunosuppressive microenvironments within the lesions. This multi‐layered regulatory framework reflects TCM principles of holistic modulation and may inform future strategies for tailoring interventions to distinct stages of tumor development.

From a broader perspective, while network pharmacology has made significant advances in elucidating the multi‐target and multi‐mechanism actions of herbal medicines (Li et al. 2023), traditional network pharmacology models remain limited in certain aspects. For instance, traditional models primarily focus on “compound‐target” binary relationships, which fail to capture critical features of TCM, such as its holistic perspective and individualized treatment strategies. These limitations are particularly evident in the inability to systematically evaluate the heterogeneity of dominant cell domains within tumor microenvironments and the dynamic changes of these domains during different disease stages. To address these challenges, our study introduces the innovative concept of “holistic regulation based on dominant cell domains,” which aims to transcend the traditional reductionist approach of focusing on single molecular targets. This framework emphasizes precise modulation of key cellular domains while highlighting the overall effects mediated by inter‐domain signaling and interactions. This analytical framework reflects the holistic perspective of TCM and conceptually parallels emerging paradigms in precision oncology. Despite uncovering significant regulatory effects of SCP in CRC lesions, this study has certain limitations. For example, interpatient variability and the potential synergistic effects of multiple SCP components require deeper investigation. More importantly, while this study identified dominant cellular domains (neutrophil‐enriched in primary CRC, macrophage‐enriched in metastases) as primary targets for SCP, the intricate interplay within the TME warrants deeper exploration. The potential mechanisms by which SCP, acting on these key domains, might indirectly regulate other critical TME components—such as CAFs, Tregs, and DCs—through intra‐domain or inter‐domain signaling crosstalk remain largely undiscussed. Future studies incorporating broader cellular interaction networks and functional validation are essential to fully elucidate these complex, indirect regulatory pathways governing the TME's response to SCP intervention. Addressing these issues will provide a more comprehensive understanding of SCP's molecular mechanisms and facilitate its clinical translation.

## 5 Conclusion

This study establishes a preliminary framework integrating spatial and single‐cell analysis with network pharmacology to explore how the TCM compound SCP may influence key inflammatory domains in CRC. SCP‐associated targets were linked to CEACAM and APP signaling in neutrophil‐ and macrophage‐enriched domains, offering insights into domain‐specific regulatory mechanisms. These findings highlight potential research directions but require further experimental validation before clinical translation can be considered.

## Author Contributions

**Yuan‐jie Liu:** conceptualization (equal), formal analysis (equal), validation (lead), visualization (lead), writing – original draft (lead). **Qian‐wen Ye:** conceptualization (equal), data curation (equal), methodology (equal), writing – original draft (equal), writing – review and editing (equal). **Yi‐lan Jin:** formal analysis (equal), investigation (equal). **Qian Zhang:** conceptualization (equal). **Xi Zou:** formal analysis (equal), project administration (lead), software (equal), supervision (equal), writing – review and editing (lead). **Jie‐pin Li:** formal analysis (equal), funding acquisition (equal), methodology (equal), writing – review and editing (equal).

## Ethics Statement

The study's protocol was approved by the ethics committee of Affiliated Hospital of Nanjing University of Chinese Medicine (Ethics approval number: 2021NL‐206‐01, Ethics approval date: 2021‐11‐19).

## Consent

Prior to the commencement of data collection, the first author obtained written consent from all participants concerning participation and subsequent publication of the study results.

## Conflicts of Interest

The authors declare no conflicts of interest. All authors contributed to data analysis, drafting, or revising of the article; agree on the journal to which the article is being submitted; provided final approval of the version to be published; and agree to be accountable for all aspects of the work.

## Supporting information


**Data S1:** fsn370794‐sup‐0001‐Supplemental Figures.docx.

**Data S2:** fsn370794‐sup‐0002‐Table S1.xlsx.

**Data S3:** fsn370794‐sup‐0003‐Table S2.xlsx.

## Data Availability

The data and materials supporting the findings of this study are available upon reasonable request. The following R packages were used for the analysis and are publicly available: clusterProfiler: https://github.com/YuLab‐SMU/clusterProfiler. ggplot2: https://github.com/tidyverse/ggplot2. AddModuleScore: https://github.com/WalterMuskovic/AddModuleScore. Seurat (v5.0): https://github.com/satijalab/seurat. SPACET (v0.1.0): https://github.com/data2intelligence/SpaCET. BayesSpace (v1.6.0): https://github.com/edward130603/BayesSpace. spatialCluster: https://github.com/mpadge/spatialcluster. SpaTopic (v0.1.0): https://github.com/xiyupeng/SpaTopic. CellChat: https://github.com/sqjin/CellChat. HPAanalyze (v1.0): https://github.com/anhtr/HPAanalyze. All links provided lead to the official repositories for these packages, where relevant documentation and usage instructions are available for further reference.
