# Supplementary material for: Spatial Domain‐Based Approach to Analyze the Mechanism of Sparganii Rhizoma‐Curcumae Rhizoma Pair in the Treatment of Colorectal Cancer
Source: Food Sci Nutr. 2025 Aug 19;13(8):e70794. doi: 10.1002/fsn3.70794 (PMC12365343; doi:10.1002/fsn3.70794)
Supplement: Supplementary file 1 — Data S1: fsn370794‐sup‐0001‐Supplemental Figures.docx. [file FSN3-13-e70794-s003.docx]

**Figure Legends**

**Figure S1**


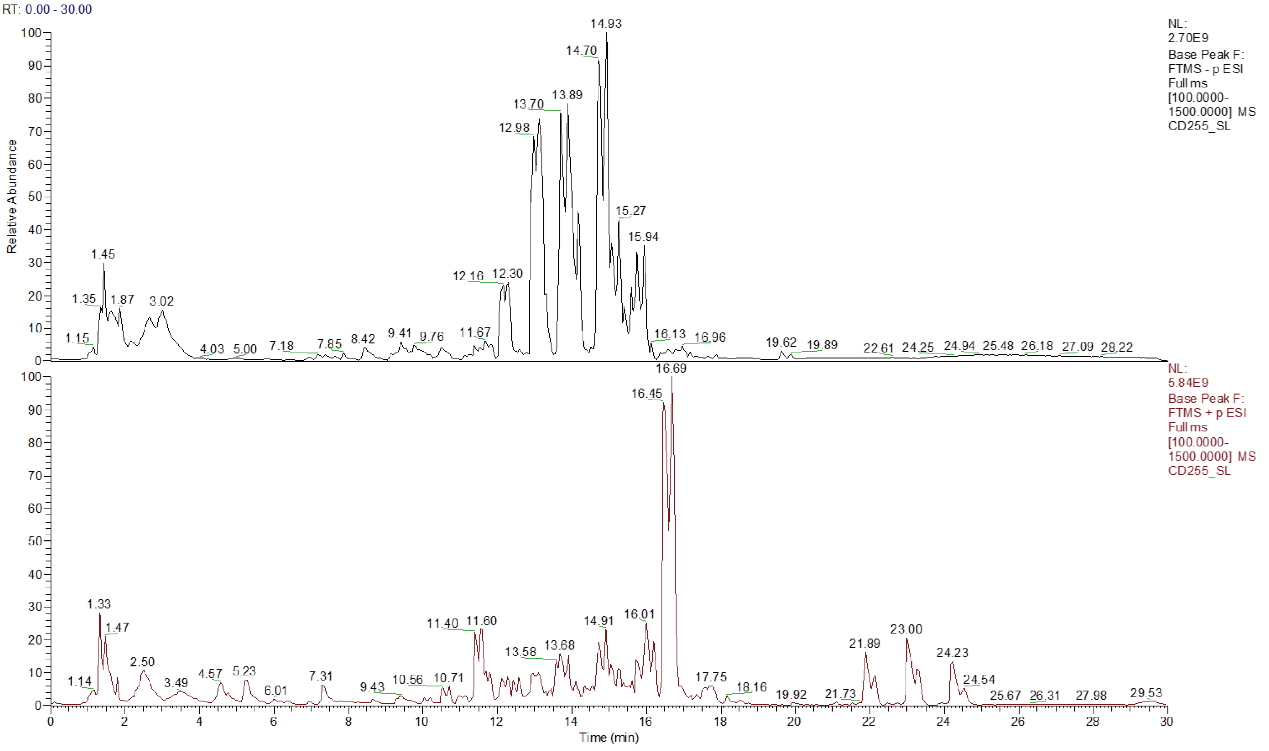


**Figure S1. Total ion chromatograms of SCP acquired in positive and negative modes.**

The total ion chromatograms (TICs) of SCP were obtained using Q-Orbitrap high-resolution mass spectrometry (HRMS). The black trace represents the negative ion mode, while the red trace represents the positive ion mode. Chromatographic separation was carried out on an AQ-C18 column (150×2.1 mm, 1.8 µm) using a mobile phase gradient of acetonitrile and 0.1% formic acid in water, with a flow rate of 0.30 mL/min. Data were acquired at a resolution of 70,000 (m/z 200) with a scanning range of 100–1500 m/z, and all analyses were performed using Xcalibur software.

**Figure S2**


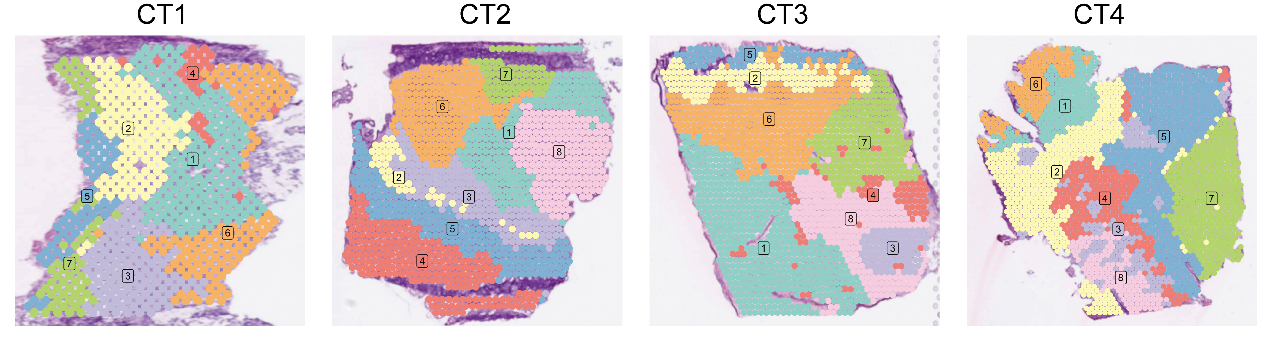


**Figure S2. Spatial domain segmentation of primary tumors (CT1-CT4) using the “BayesSpace” R package.**

Spatial domains of primary tumors (CT1-CT4) were identified using the BayesSpace R package, which applies Bayesian modeling to spatial transcriptomics data. The resulting spatial map illustrates distinct domains delineated by different colors, each representing regions with unique transcriptional profiles.

**Figure S3**


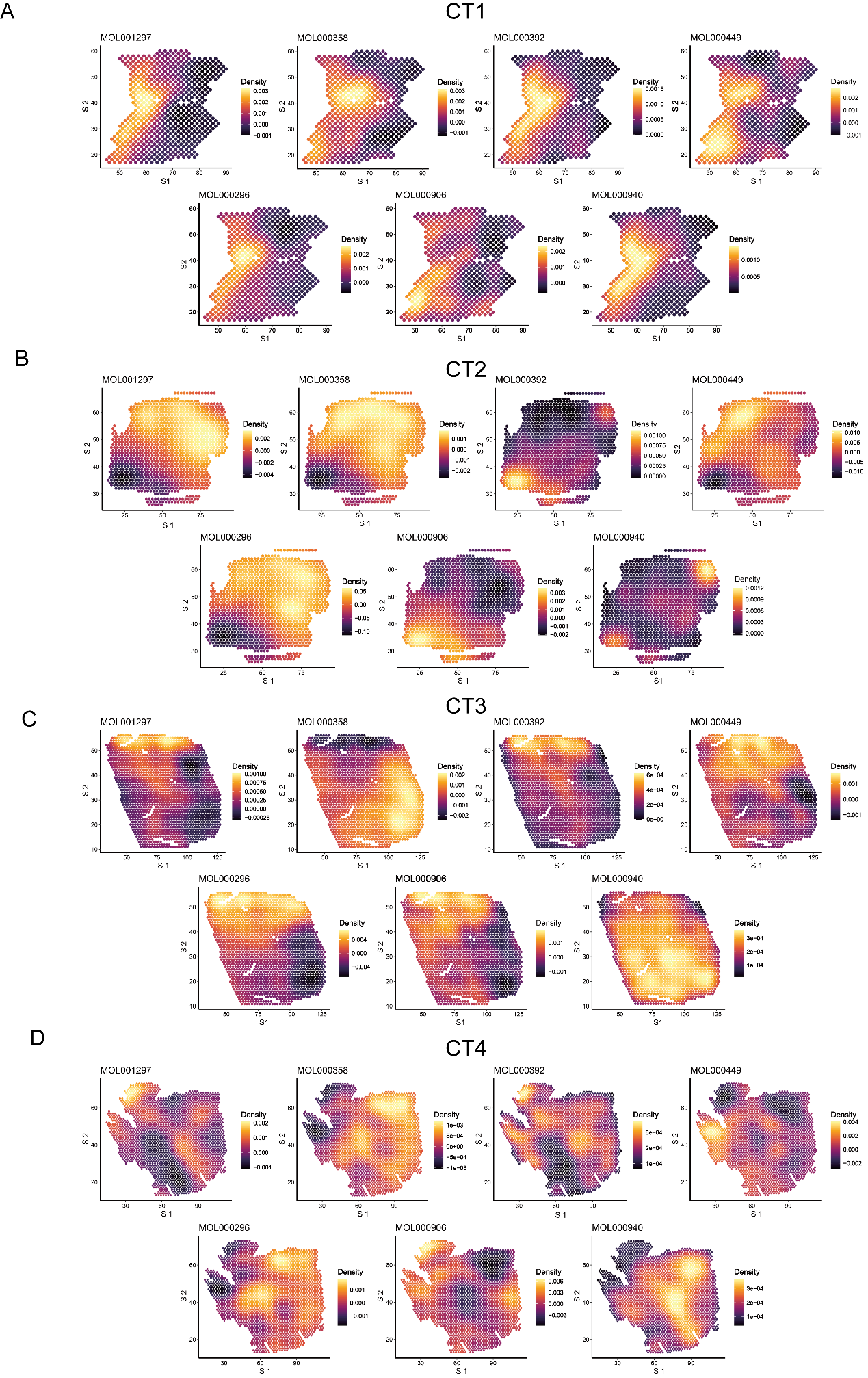


**Figure S3. Spatial targeting of SCP core components to distinct cellular domains in four primary tumors (CT1–CT4)**.

(A-D) The spatial mapping reveals distint cellular domains within four primary tumors (CT1–CT4) that are targeted by SCP core components. Brighter colors indicate stronger targeting intensity in specific domains, highlighting functional hotspots of SCP activity.

**Figure S4**


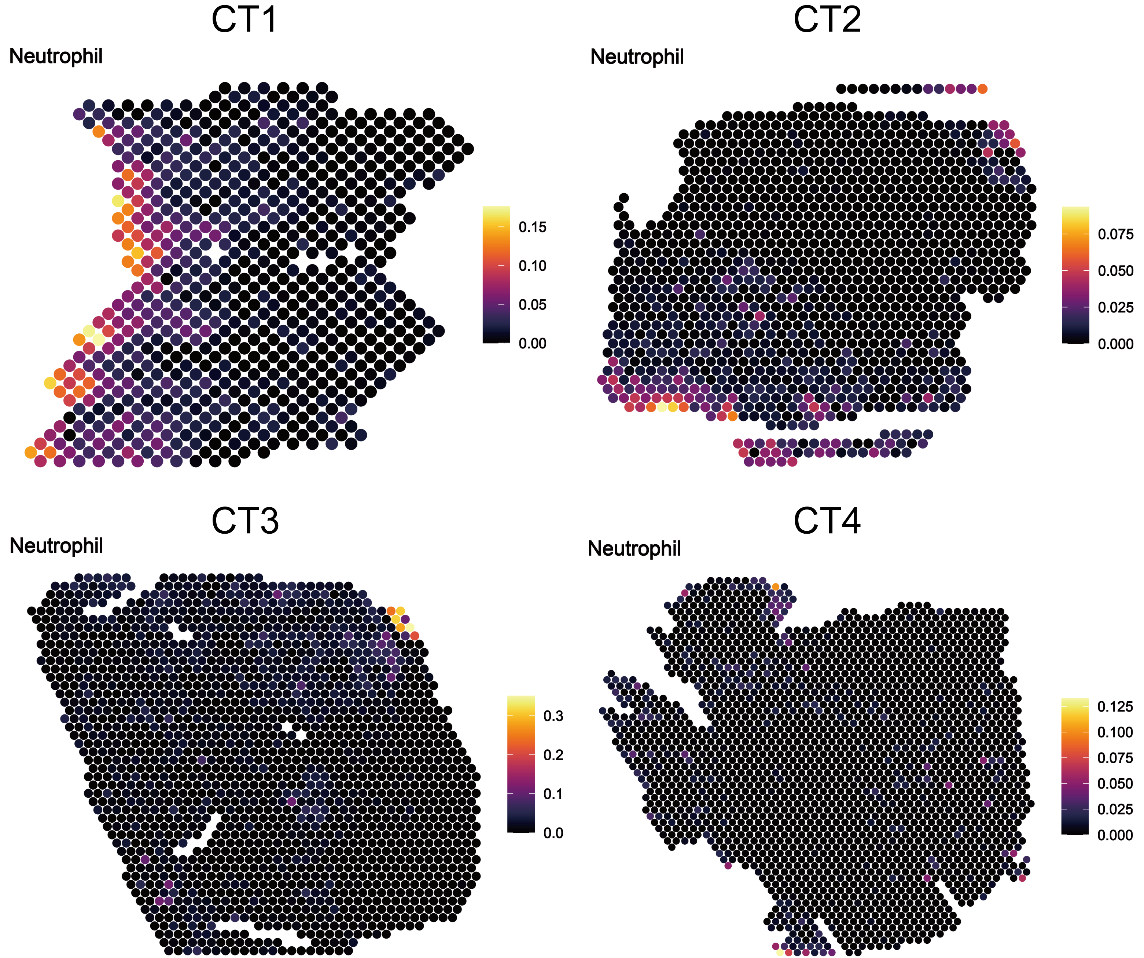


**Figure S4. Spatial localization of neutrophils in four primary tumors (CT1–CT4).**

The spatial positioning of neutrophils was analyzed in four primary tumors (CT1–CT4) using spatial transcriptomics. Neutrophil-enriched regions are identified and represented by distinct spatial domains, with color intensity corresponding to the density of neutrophil-associated gene expression.

**Figure S5**


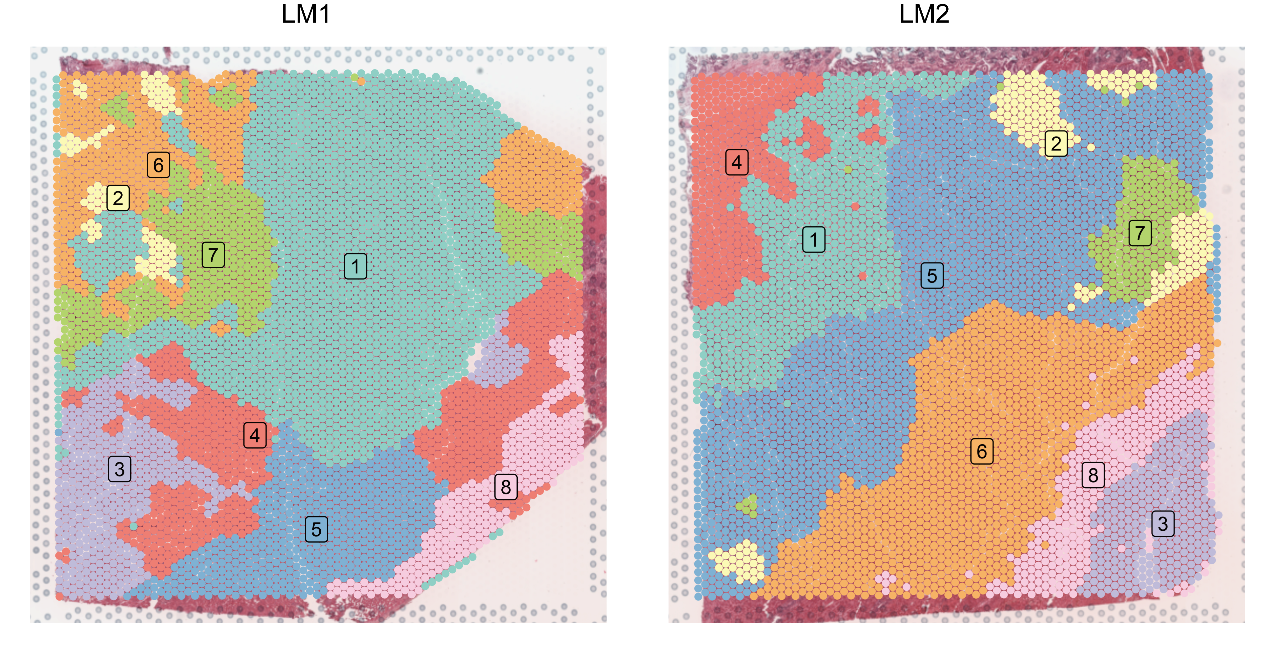


**Figure S5. Spatial domain segmentation of liver metastases (LM1 and LM2) using the “BayesSpace” R package.**

Spatial domains of liver metastases (LM1 and LM2) were identified using the “BayesSpace: R package, which applies Bayesian modeling to spatial transcriptomics data. The resulting spatial map illustrates distinct domains delineated by different colors, each representing regions with unique transcriptional profiles.

**Figure S6**


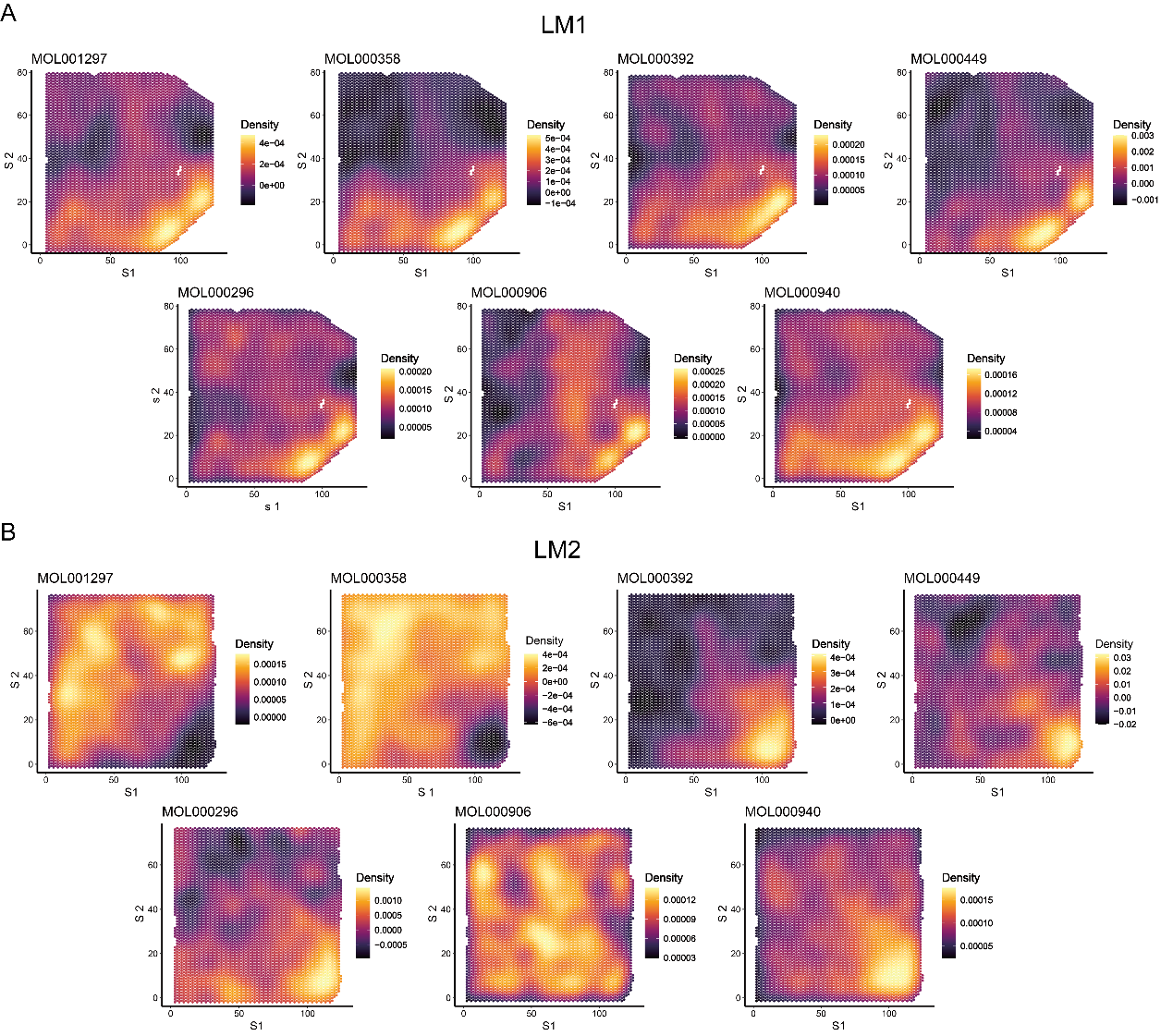


**Figure S6. Spatial targeting of SCP core components to distinct cellular domains in two liver metastases (LM1 and LM2).**

(A, B) The spatial mapping reveals distinct cellular domains within two liver metastases (LM1 and LM2) that are targeted by SCP core components. Brighter colors indicate stronger targeting intensity in specific domains.

**Figure S7**


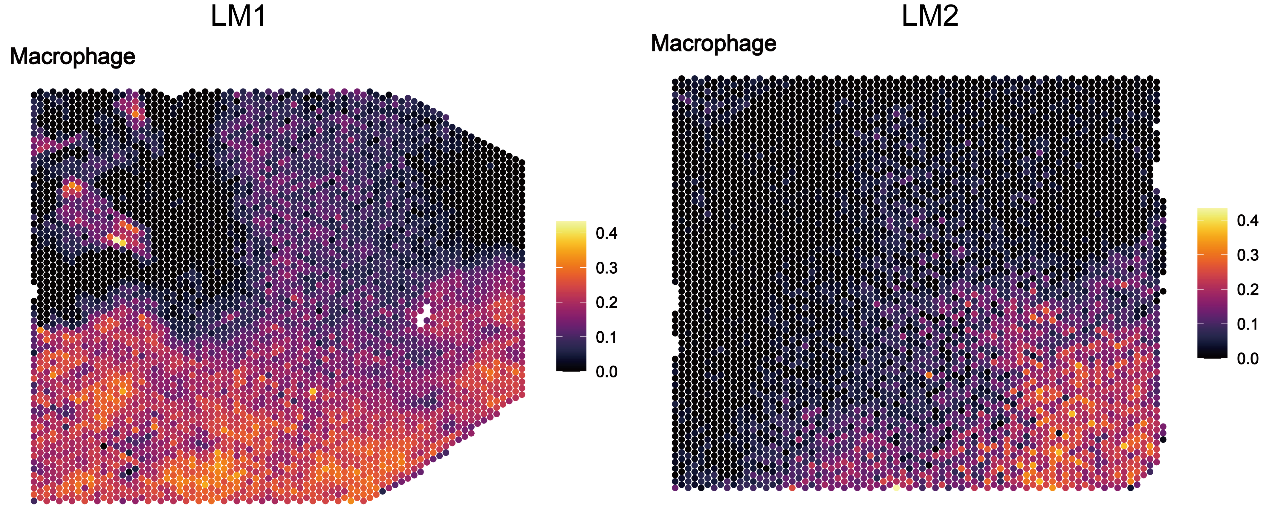


**Figure S7. Spatial localization of macrophages in two liver metastases (LM1 and LM2).**

The spatial positioning of macrophages was analyzed in two liver metastases (LM1 and LM2) using spatial transcriptomics. Macrophage-enriched regions are identified and represented by distinct spatial domains, with color intensity corresponding to the density of macrophage-associated gene expression.
